# Non-visual hallucinations in Parkinson’s disease: a systematic review

**DOI:** 10.1007/s00415-022-11545-6

**Published:** 2023-01-27

**Authors:** Wei Lin Toh, Caitlin Yolland, Caroline Gurvich, James Barnes, Susan L. Rossell

**Affiliations:** 1 Centre for Mental Health and Brain Sciences, School of Health Sciences, Swinburne University of Technology, Melbourne, PO Box 218, Hawthorn, 3122 VIC Australia; 2 Department of Psychiatry, St Vincent’s Hospital, Melbourne, VIC Australia; 3 Department of Psychology, Alfred Hospital, Melbourne, VIC Australia; 4 HER Centre, Monash University Central Clinical School, The Alfred Hospital, Melbourne, VIC Australia; 5 Department of Psychology, Fatima College of Health Sciences, Abu Dhabi, UAE

**Keywords:** Parkinson’s disease, Auditory hallucinations, Olfactory hallucinations, Somatic-tactile hallucinations, Phenomenology

## Abstract

**Background:**

Non-visual hallucinations in Parkinson’s disease (PD) can be prevalent and distressing. Most existing research has however, focused on visual hallucinations as well as related risk factors. The current study thus conducted a systematic review to collate existing evidence on non-visual hallucinations in PD, focusing on their prevalence, phenomenology, and clinical-cognitive correlates.

**Methods:**

Ninety-one relevant studies were included from a systematic search across PsycINFO APA, PubMed, and Web of Science, for peer-reviewed publications in the English language, from 1970 to the present. These comprised a mix of case (30 studies; *n* = 56) and group design (62 studies; *n* = 7346) studies, divided into three somewhat overlapping collections to address our three research foci.

**Results:**

Prevalence estimates for hallucinations were: auditory 1.5–72.0%, olfactory 1.6–21.0%, somatic-tactile 0.4–22.5%, gustatory 1.0–15.0%, and sensed presence 0.9–73.3%. Phenomenological inquiries revealed descriptions of vivid, consuming events replete with elaborate detail, adversely affecting PD patients in different ways. Overt experiences of multisensory hallucinations were also highly variable (0.4–80%) but exceedingly common, reported by almost half of the 45 included prevalence studies. There was some evidence for modality-specific hallucination predictors, but this was largely tentative, pending robust replication.

**Conclusions:**

Marked prevalence figures coupled with phenomenological descriptions implicating distress denote that non-visual and multisensory hallucinations in PD are of clinical significance. More direct research and clinical attention need to be devoted to the study and management of such hallucinatory experiences.

**Supplementary Information:**

The online version contains supplementary material available at 10.1007/s00415-022-11545-6.

## Introduction

Parkinson’s disease (PD) is a neurodegenerative disorder characterised by prominent motor and non-motor symptoms [1]. Classification as a movement disorder derives from its disabling motor symptoms, such as a resting tremor, bradykinesia, and rigidity, but non-motor symptoms also exert significant impact [2]. Hallucinations are one such symptom, typically regarded as belonging to the psychiatric realm, but can be prevalent and distressing [3, 4]. Another non-motor symptom relates to cognitive decline; 31% of PD patients were diagnosed with dementia [5], with 83% eventually transitioning over 20 years [6]. In fact, presence of visual hallucinations has been identified as a significant risk factor for developing dementia [7, 8]. Yet beyond the visual mode, scant attention has been given to hallucinatory experiences in other sensory modalities in PD as well as their potential clinical and cognitive correlates.

### Hallucinations in PD: prevalence, phenomenological characteristics and correlates

With the publication of a consensus definition of psychosis in PD, clinical and research attention to the role of hallucinations in the disorder has intensified [9]. An early epidemiological review reported point prevalence of up to 38% visual hallucinations (lifetime 50%) and 22% auditory hallucinations in PD, observing that olfactory and somatic-tactile hallucination rates were not systematically noted [10]. A comparative review of visual hallucination incidence across Parkinsonism disorders instead cited rates up to 75% [11]. A more recent meta-analysis calculated pooled point prevalence of 28% and 9% for visual and auditory hallucinations respectively [12]; whereas a separate review noted 25% for sensed presence [13]. These authors concluded that hallucinations tended to increase and worsen over time [10], and highlighted that use of validated assessment tools yielded elevated estimates [12].

Detailed phenomenological descriptions of visual hallucinations in PD are plentiful. These include complex images of variable content, but often comprise familiar or unfamiliar people, or animals [14–18]. Frequency of visual hallucinations has varied from numerous times a day to weekly or less, most likely in the evening or at night, experienced as kinetic and stereotyped [15], typically of brief duration lasting seconds to minutes, with no specific localisation [14], and were somewhat blurred, with no known triggers [18]. Though non-threatening themes have been regularly endorsed [15], visual hallucinations could be perceived as emotionally neutral [14], or unpleasant and anxiety-provoking [16]. Conviction has similarly been mixed [14] or preserved, yet involving complex interactions, such as inviting ‘guests’ to dinner, or pushing an unwanted companion away [17]. Visual hallucinations have occurred in isolation, or in conjunction with other hallucinations or delusions [15]. For instance, comorbid visual and tactile perception of hairs on an arm, or lice and worms burrowing into the skin, have been described, with such multisensory events often perceived as distressing [17]. Conversely, phenomenological information regarding auditory hallucinations in PD has received limited attention. Most common were voices perceived as originating outside the head, often incomprehensible or indistinct [12], or familiar, and even persecutory on occasion [15]. Auditory hallucinations have sometimes been described as providing a “soundtrack” to visual hallucinations, for example, hearing people in a vision conversing [14]. Non-verbal sounds have also been reported, but musical hallucinations were deemed rare [12]. Inclusive descriptions of hallucinatory experiences across other sensory modalities were generally lacking, but tactile hallucinations have been cursorily described as the feeling of being touched by a person, or involving contact with small animals [14].

Cognitive decline is often seen in PD [5, 6], with cognitive impairment and visual hallucinations described as risk factors for mutual emergence [19]. Increased incidence of visual hallucinations during cognitive decline has been noted, with these experiences reported as qualitatively different, inciting combative behaviours, and exacerbated by lack of insight [17]. Several reviews have shown that PD patients with visual hallucinations exhibited significantly poorer cognition overall as well as within specific domains, involving attention, executive function, memory, and visual processing [20–22]. Those with visual hallucinations were also troubled by more severe clinical symptoms, such as greater duration and severity of PD illness, and depressed mood [18]. Alongside older age and sleep disturbances, all these factors have been noted as significant predictors of (visual) hallucinations in PD [10].

Other than these disease-related factors, there has also been scrutiny around possible neurophysiological underpinnings of visual hallucinations in PD. These have ranged from structural atrophy and/or aberrant activation across multiple brain regions, including frontal and visual cortices, genetic and familial origins or even the potential contribution of dopaminergic or anticholinergic therapies [10, 14, 18, 20, 23]. Some authors have attempted to offer up integrative theoretical models seeking to explain the rise and progression of visual hallucinations in PD [24, 25], though more work remains to be done. Likewise, visual hallucinations tend to be reported in other neurodegenerative (e.g. dementia with Lewy bodies), or ophthalmological (e.g. Charles Bonnet syndrome) disorders, though wide-ranging prevalence estimates make it difficult to draw any firm conclusions about relative frequencies [26–28]. In contrast, studies delving into non-visual or multisensory hallucinations in PD, including their neurophysiological causes, are notably lacking.

### Aim and research questions

Based on a brief review of the literature, it is evident that hallucinations studies in PD have focused on the visual realm. Reviews have covered topics ranging from aetiological models [25] to neuroanatomical correlates [29] and management strategies [30] for visual hallucinations, but less so in other sensory modalities. Even studies broadly mentioning hallucinations were often simply referring to those in the visual domain. Yet hallucinations are not a unitary construct, encompassing a complex, multifaceted array of discrete yet overlapping perceptual experiences. Discrimination of modality-specific hallucinations in PD may aid prognostic and therapeutic outcomes. However, we first need to better understand these experiences.

The aim of the current study was to examine non-visual and multisensory hallucinations in PD, with the latter referring to experiences in two or more sensory modalities (often but not always including visual), concurrent or otherwise. The following research questions were put forward:

(i) What was the prevalence of non-visual (i.e. auditory, olfactory, somatic-tactile, gustatory, and sensed presence) and multisensory hallucinations in PD?
(ii) What were the phenomenological characteristics of these hallucinatory experiences, including physical (e.g. frequency, duration, intensity), cognitive (e.g. controllability, conviction, interaction) and emotional (e.g. valence, distress, functional impact) facets?
(iii) What were the key clinical and cognitive correlates underlying the occurrence of these modality-specific hallucinations in PD? In line with existing research [10], clinical correlates of interest were participant age, age of PD diagnosis, length of illness, PD severity, and presence of depression. Cognitive correlates included presence of dementia or cognitive decline (general or domain-specific).

## Methods

The review adhered to guidelines described in the Preferred Reporting Items for Systematic Reviews and Meta-Analyses (PRISMA) statement [31]. The study protocol was pre-registered on the International Prospective Register of Systematic Reviews (PROSPERO; CRD42019124981). The authors confirm that the approval of an institutional review board and patient consent was not required for this work. We confirm that we have read the Journal’s position on issues involved in ethical publication and affirm that this work is consistent with those guidelines.

### Search strategy

**Fig. 1:**
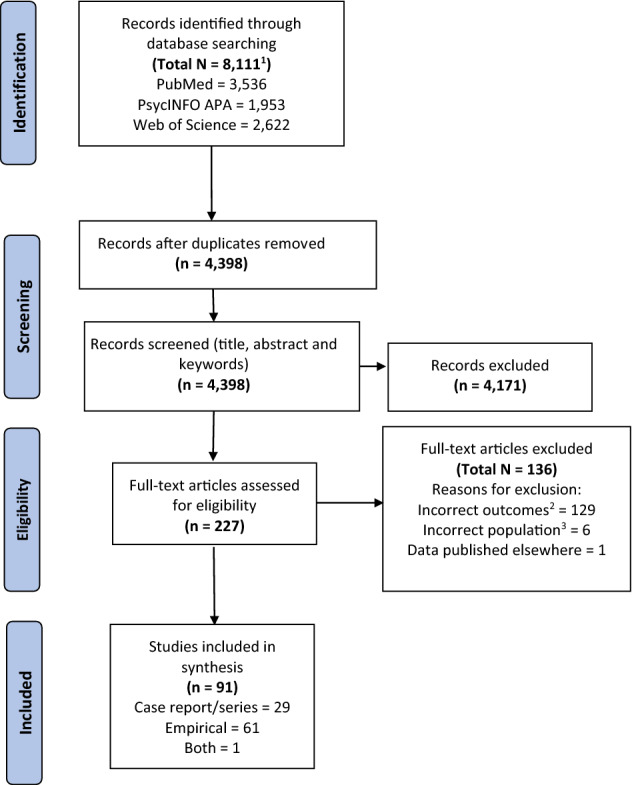
PRISMA flow diagram ^1^Search cut-off date: 15/02/2022 ^2^Incorrect outcomes included: **a** Non-modality-specific hallucinations *n* = 91; **b** Limited phenomenological information *n* = 30; and **c** Main outcome not prevalence, phenomenology or correlates *n* = 8.^3^Pertaining to or combined with other neurological disorder(s)

A flowchart illustrating the search strategy and study selection is presented in Fig. 1. A systematic search was conducted across PsycINFO APA, PubMed, and Web of Science, for peer-reviewed publications in the English language, from 1970 to the present, with a cut-off date of 15 February 2022. Search syntax was optimised for each database, centred on three dominant themes: PD, non-visual or multisensory hallucinations, as well as key clinical and cognitive correlates. These terms (and their permutations) were entered into a search matrix using Boolean operators to ensure pertinent combinations were incorporated (see Table A in Supplementary materials for the search syntax). Reference lists of eligible studies were cross-checked for further related works.

### Study selection

After duplicates were removed, the title, abstract and keywords of retrieved publications were screened by CY, where irrelevant studies were excluded. A fidelity check was conducted where WLT also independently reviewed the first 100 abstracts, yielding an excellent interrater reliability of *k* = 0.852 (i.e. complete agreement for 99/100 papers, with CY taking the more conservative stance of screening in one additional paper). This corroborated that the screening approach undertaken by CY for the remaining abstracts was targeted and robust. Full-text articles were then independently reviewed by WLT and CY for eligibility, and included if they comprised case report or group design studies investigating the prevalence, phenomenology and/or correlates of non-visual or multisensory hallucinations in PD. For intervention studies, only baseline data was used (if relevant); longitudinal studies were also permitted. Studies focusing solely on visual (or non-modality-specific) hallucinations were excluded (*n* = 91; nb. this inflated figure provides further support showing how non-visual hallucination studies have been neglected in lieu of those focusing on visual hallucinations), and unpublished data were not pursued. Discrepancies in study inclusion between CY and WLT were resolved through iterative discussions with senior author SLR, until consensus was achieved.

### Data extraction and risk of bias assessment

Data extraction was jointly performed by WLT and CY, and comprised: Study identifiers (e.g. authors, year of publication and aims), participant characteristics (e.g. age, sex, subgroup sizes), prevalence data (e.g. percentages of non-visual or multisensory hallucinations), phenomenological descriptions (e.g. physical, cognitive and emotional facets of hallucinatory experiences), as well as key clinical and cognitive correlates (e.g. length and severity of PD illness, general and domain-specific cognition). Where information pertaining to visual hallucinations was presented alongside that for non-visual hallucinations, it was included in data extraction. This approach ensured completeness, especially critical for phenomenology, though extracted data for visual hallucinations should be interpreted with caution in light of this. To avert replication for multiple publications using the same cohort, the study with the most representative data aimed at answering our research questions was included; studies employing similar datasets were included only if distinct aspects of the empirical data were analysed.

The search strategy yielded a total of 8111 records, from which 91 case or group design studies met inclusion criteria. These studies were divided into three somewhat overlapping collections to address our three research foci, namely: (i) prevalence (*n* = 45), (ii) phenomenology (*n* = 56), and (iii) key clinical and cognitive correlates (*n* = 16). To fulfil a more stringent consideration of prevalence, smaller-scale studies where *n* ≤ 50 participants were removed from the first collection (*n* = 17 studies; see Table B in Supplementary materials). Likewise, studies focusing on overall hallucinations (i.e. not modality-specific) were omitted from the third collection (*n* = 15; see Tables C and D in Supplementary materials). Owing to the nature of our research questions, the first and third collections considered group design studies only, whereas the second collection encompassed case and group design studies. The varied nature of data extracted meant that a narrative synthesis of findings is provided.

A risk of bias assessment was conducted by CY using the JBI Critical Appraisals Checklist for Case Studies [JBI-CAC, supplemented by one additional item from the corresponding checklist for Case Series; [32] and Newcastle–Ottawa Quality Assessment Scale for Case Control Studies [NOQAS; [33] for case and group design studies respectively, adapted for our purpose (see Tables E and F in Supplementary materials). Within each of the six domains, 0–2 points may be awarded based on how well each response satisfied the criterion under assessment (nb. only 0–1 point may be awarded for *representativeness* and *comparability* in the NOQAS). Unweighted summed scores provided an overall measure of quality and bias. Categorical ratings were devised where: *Excellent* 10–12/12, *good* 7–9/12, *fair* 4–6/12 and *poor* ≤ 3/12 for JBI-CAC; and *Excellent* 9–10/10, *good* 7–8/10, *fair* 5–6/10 and *poor* ≤ 4/10 for NOQAS).

## Results

### Prevalence of multisensory hallucinations in PD

Prevalence data are presented in Table 1, where 6968 PD patients across 45 studies were included. Of these, a fraction was further classed as having dementia (*n* = 48) and/or psychosis (*n* = 319). Prevalence of overall hallucinations ranged from 12.8 to 100% (54.2% and 77.1–100% respectively for those with dementia or psychosis in PD), whereas that for multisensory hallucinations ranged from 0.4 to 80% (modalities not specified). Within each primary sensory domain, these figures were: auditory 1.5–72%, olfactory 1.6–21%, somatic-tactile 0.4–22.5%, gustatory 1.0–15.0%, and sensed presence 0.9–73.3% (visual 3.0–96.9%). Where available, we also extracted prevalence data for hallucinations in a single modality (see Table 1). Given the small proportions of PD patients classed as having dementia and/or psychosis, it was difficult to draw any firm conclusions as to whether prevalence estimates across single hallucination modalities appeared to differ in typical versus atypical PD. When interpreting these wide ranges, it is imperative to pay heed to the specific topic examined within each study, as this would have influenced the corresponding figures in some way (e.g. a study focused on sensed presence would likely have recruited a surfeit of participants experiencing this phenomenon, neglecting other hallucinatory experiences). On this note, focusing explicitly on studies that named prevalence as their predominant aim (*n* = 6), more precise point prevalence rates of 12.8–39.8% for overall (46.3% lifetime), and 2.1–10.3% for olfactory hallucinations as well as 23% for sensed presence were recorded (14.3% for visual). No existing studies specifically aimed to examine the prevalence of auditory, somatic-tactile or gustatory hallucinations in PD. It is noted that the sporadic and inconsistent nature of figures extracted across the sensory domains precluded a more systematic approach to data summation, including use of meta-analysis or forest plots, in a meaningful way.

**Table 1:** Prevalence of multisensory hallucinations in Parkinson’s disease (*n* = 45 studies)

Author, year	Topic in PD	Participants; subgroups	Prevalence (%)							
			Any hall	MSH	VH	AH	OH	TH	GH	SP
Aarsland et al., 2001 [66]	Psychiatric symptoms	131; PD = 83, PD_dementia_ = 48	29.0	–	23.7	12.2	3.1	0.8	–	–
Bannier et al., 2012 [40]	Prevalence of OH	87	–	VH + OH = 2.3, VH + AH + OH = 3.4	–	–	10.3, OH_only_ = 4.6	–	–	–
Barrett et al., 2017 [67]	Psychosis (without dementia)	101; PD_psychosis_ = 33	19.8; 60.6	VH + = 6.9	12.9	7.9	4.0, OH_only_ = 3.0	2.0, somatic_only_ = 1.0	–	5.0, SP_only_ = 1.0
Chendo et al., 2021 [68]	Psychosis in PD	92; PD_psychosis_ = 51	52.2; 94.1	–	48.9	17.4	–	–	–	15.2
Chou et al., 2005 [57]	DIP	160	98.1	VH + = 56.3	96.9, VH_only_ = 40.6	47.5	15.6	22.5	–	–
de Chazeron et al., 2015 [43]	Hall scale validation	86	100	OH + GH = 14.0	86.0	41.9	na	cenesthetic = 12.8	–	73.3
de Maindreville et al., 2005 [69]	Longitudinal hall	127–141	41.7–49.6	–	21.3–22.8	8.7	–	–	–	29.1–40.2 (MH)
Factor et al., 2014 [70]	Cognitive correlates of psychosis	144; PD_psychosis_ = 48	25.7; 77.1	–	14.6, VH_only_ = 9.0	11.8, AH_only_ = 6.3	4.9	5.6	–	–
Fenelon et al., 2000 [48]	Prevalence and risk factors of hall	216	39.8, 46.3 (lifetime)	VH + = 57.9	22.2, VH_only_ = 9.3	9.7, AH_only_ = 2.3	–	–	–	16.2, MH_only_ = 13.9
Fenelon et al., 2011 [35]	SP	130	–	FP + = 24.6; SP + VH = 15.4, FP + AH = 10.8, FP + OH = 6.9, FP + TH = 10.8, FP + GH = 3.8	–	–	–	–	–	40
Fenelon et al., 2010 [61]	Psychosis	116	42.2	–	16.4	18.1	11.2	12.1 (somatic = 0.9)	2.6	32.8
Goetz et al., 2011 [38]	10-year longitudinal VH and non-VH	60_baseline_ → 24_follow-up_	13.0_baseline_ → 63.0_follow-up_	VH + = 0–60.0; VH + AH = 20.0, VH + OH = 6.7, VH + TH = 6.7, VH + AH + TH = 26.7	VH_only_ = 88.0_baseline_ → 33.0_follow-up_	72.0, AH_only_ = 57.0	21.0, OH_only_ = 36.0	48.0, TH_only_ = 7.0	–	–
Goetz et al., 1998 [58]	DIP	70	–	VH + = 22.9	100.0	18.6	7.9	14.3	–	–
Holroyd et al., 2001 [71]	Hall and del	102	29.4 (del included)	VH + AH = 2.0, VH + GH = 1.0	25.5	–	–	–	–	–
Ikeda et al., 2016 [72]	3-year longitudinal of hall and fatigue	78_baseline_ → 63_follow-up_	–	VH + AH = 9.5	49.2 (first onset)	–	–	–	–	–
Inzelberg et al., 1998 [39]	AH	121	37.2	VH + AH = 8.3	VH_only_ = 28.9	AH_only_ = 0	–	0	–	–
Kataoka and Ueno, 2015 [36]	Risk factors for FP	78_baseline_ → 58_follow-up_	–	SP + = 20.7; SP + VH + AH = 6.9	–	–	–	–	–	SP_only_ = 8.6 (first onset)
Katzen et al., 2010 [56]	MSH and cognition	152	–	VH + = 7.9	30.9, VH_only_ = 23.0	–	–	–	–	–
Kulick et al., 2018 [46]	MSH, SP and del	199	28.1	2.5	3.0	1.5	5.5	3.5	1.0	6.0
Lee and Weintraub, 2012 [73]	Psychosis (without dementia)	191	–	VH + AH = 1.6	13.6	6.8	–	–	–	–
Lenka et al., 2017 [74]	Predictors of psychosis	51	–	VH + AH = 3.9, OH + TH = 1.9	VH_only_ = 10.4	AH_only_ = 1.9	–	1.9	–	–
Leu-Semenescu et al., 2011 [75]	Hall and sleep	100	26.0	8.0	26.0	2.0	–	4.0	–	15.0 (MH)
Llorca et al., 2016 [44]	MSH	100		80.0	88.0	45.0	15.0 (or GH)	Cenesthetic = 14.0	15.0 (or OH)	70.0
Mack et al., 2012 [76]	Prevalence of psychotic symptoms	250	12.8	–	6.8	3.6	2.0	1.2	–	3.6
Marsh et al., 2004 [77]	Psychiatric comorbidities in psychosis	116; PD_psychosis_ = 25	19.8; 92.0	–	16.4	12.9	2.6	3.4	–	0.9
Matsui et al., 2007 [41]	Thalamic perfusion in VH	83	–	VH + AH = 3.5	VH_only_ = 44.6	7.2	–	–	–	–
McAuley and Gregory, 2012 [78]	Prevalence of OH	188	–	–	15.4	2.1	2.1	–	–	–
Muller et al., 2018 [79]	VH scale validation	163	–	–	19.6	14.7	9.2	8.0	3.7	21.5
Nishio et al., 2017 [80]	Psychosis	67	22.4	VH + AH = 7.5	VH_only_ = 17.9	AH_only_ = 0	–	–	–	23.9
Omoto et al., 2021 [81]	Risk factors for SP	100	–	–	17.0	5.0	2.0	3.0	–	32.0
Pacchetti et al., 2005 [82]	Psychosis and sleep	289	29.8	VH + AH = 5.2; VH + TH = 1.4	29.8, VH_only_ = 23.2	2.8	–	–	–	–
Paleacu et al., 2005 [83]	Hall and familial dementia	234	32.5	VH + AH = 2.6, VH + TH = 0.4	32.5	2.6	–	0.4	–	–
Papapetropoulos et al., 2008 [84]	Hall scale validation	70	44.3	MSH = 15.7; VH + AH = 7.1	34.3, VH_only_ = 20.0	17.1, AH_only_ = 5.7	7.1, OH_only_ = 2.9	Somatic = 2.9	1.4	–
Perez-Perez et al., 2016 [62]	MSH (“string” hall)	164	–	VH + TH = 4.3 VH + AH + TH = 0.6	–	–	–	–	–	–
Rai et al., 2015 [85]	Psychiatric comorbidities (without dementia)	126	23.8	VH + AH = 7.2	20.6	7.2	1.6	Somatic = 13.5	–	–
Rana et al., 2013 [47]	Predictors of VH treatment-seeking	334	–	MSH = 0.9; VH + TH = 0.9	10.5	AH_only_ = 0.9	–	–	–	–
Sawada et al., 2013 [86]	Risk factors for psychosis	192; PD_psychosis_ = 52	–	–	17.2	1.6	–	0.5	–	–
Shine et al., 2015 [87]	Psychosis scale validation	197; PD_psychosis_ = 86			20.8	14.7	9.1	8.1	3.0	21.3
Solla et al., 2021 [34]	Frequency of OH	141	–	VH + OH = 6.4, OH + GH = 2.8	17.7	4.3	11.3	–	2.8	–
Svetel et al., 2012 [88]	Hall (without dementia)	180; PD_psychosis_ = 24	13.3; 100	VH + AH = 2.8, VH + OH = 0.6	12.2, VH_only_ = 8.9	–	–	–	–	–
Trosch et al., 1998 [60]	*Clozapine* treatment	172	–	–	66.3	5.2	–	–	–	–
Wood et al., 2015 [37]	SP	414	–	VH + SP = 8.7 AH + SP = 1.4 TH + SP = 2.7	15.5, VH_only_ = 2.2	–	–	–	–	SP = 50.2, SP_only_ = 36.7
Zhang et al., 2021 [89]	SP	149	20.1	–	12.1	9.4	8.1	4.7	–	13.4, SP_only_ = 4.7
Zhong et al., 2021 [90]	Prevalence and risk factors for SP	262	17.2	–	–	–	–	–	–	23.0
Zhu et al., 2017 [91]	Prevalence and risk factors for VH	371	–	VH + AH = 4.0	14.3	4.0	–	–	–	–

*PD* Parkinson’s disease; *DIP* drug-induced psychosis; *Hall* hallucinations; *Del* delusions; *na* not available; *MSH* multisensory hallucinations, broken down into modalities where available; *VH* visual hallucinations; *AH* auditory hallucinations; *OH* olfactory hallucinations; *TH* tactile hallucinations; *GH* gustatory hallucinations; *SP* sensed presence, also referred to as “Guardian angel”, “feeling of presence” or “extracampine hallucinations”; *MH* minor hallucinations, including SP, passage hallucinations, visual illusions and other similar phenomena; MH figures cited only when a study has not specifically provided SP rates

### Phenomenology of non-visual and multisensory hallucinations in PD

Descriptive phenomenology of non-visual and multisensory hallucinations in PD is shown in Table 2, where 1093 patients across 56 studies were included. To contextualise these findings, we opted to present participant characteristics, comprising age, sex (and number of participants for group design studies), length of illness as well as neurological and psychiatric comorbidities, where available. Hallucinatory experiences in single as well as multiple modalities were reported, spanning a range of non-visual (and visual) domains. For specific phenomenological parameters, there did not appear to be consistent patterns in how these diverse events were experienced by individual PD patients, whether in terms of their physical (e.g. frequency, duration, localisation and time of occurrence), cognitive (e.g. conviction, controllability and compliance) or emotional (e.g. content, valence, distress and functional impact) characteristics. Nor did these experiences seem to relate to participant characteristics (e.g. age) in a systematic manner. A major conclusion that may be drawn is that many of these experiences, regardless of hallucination modality, appeared to be vivid, consuming events replete with elaborate and possibly idiosyncratic detail, seemingly affecting a range of PD patients in different ways. Overt experiences of multisensory hallucinations were exceedingly common, described by almost half (48.2%) of included studies (though this could represent underreporting, given the question was mostly not explicitly asked).

**Table 2:** Phenomenology of multisensory hallucinations in Parkinson’s disease (*n* = 56 studies)

Author, year	Participant age in years^a^, sex (*n*)^b^	Length of PD illness (LOI) in years^a^	Neurological/ psychiatric comorbidities	Hallucination modality	Description of hallucination phenomenology^c^
Abe et al., 2016 [92]^d^	81 m	14	None	MSH (VH + TH)	Insects or worms invading urethra and anus causing constant pain, worst in left leg and inguinal region. Pain described as ‘worm-crawling sensation’, and only occurred in context of VH
Amar et al., 2014 [93]	54.2 ± 11.6, 88% male, 40	6.5 ± 4.5	Psychosis	VH, AH, OH, TH	Simple (8%) or complex (92%) images of familiar persons (72%), strangers (16%), or animals (12%), several times per day (76%) or per week (24%). Reactions were to ignore (48%), act out (28%), talk to (12%), or feel fearful (4%), with poor insight (52%). Simple (22%) or complex (78%) sounds of familiar persons (61%), strangers (11%), or animals (28%), such as bullets or dogs barking, several times per day (67%) or per week (33%). Reactions were to ignore (61%), act out (28%), talk to (11%), or feel fearful (0%), with poor insight (39%). Smelt oils and spices (3%). Felt contact with insects (5%) and/or small animals (5%), or snake crawling on leg (3%). Stereotyped (89%) and perceived as non-threatening (89%)
Arnulf et al., 2000 [94]^d^	69 m	7	RBD; mood disorders and cognitive impairment absent	VH, AH, OH, TH	Saw children sitting alongside on sofa, tiny people moving on wallpaper, and doll making faces; heard neighbours whispering insults; smelt unpleasant odours in bed; felt shoulder being hit
Bannier et al., 2012 [40]	65.1 ± 1.7, 33% male, 9	7.2 ± 4.1	TBI, severe cognitive impairment excluded	OH (plus VH and/or AH in some cases)	Once or twice per month, at any time of day except night, lasting seconds to minutes, related to burning, gas, or rotten food, described as bitter or unpleasant, but could also involve flowers. Perceived as not frightening, with low disruption to function, and good insight
Benbir et al., 2006 [95]	66.7 ± 8.9, 64% male, 70	8.5 ± 4.8	MDD (9%); dementia or psychosis excluded	VH, AH, TH	Brief images of familiar or unfamiliar people or animals. Unidentified sounds as well as voices of deceased relatives or unfamiliar persons. Felt invisible person’s touch, or bugs moving on skin. Good (47%) or poor (53%) insight
Clark, 1998 [96]^d^	78 f	0	No hearing loss or tinnitus; mild anxiety and MDD	AH (musical)	Heard popular songs inside head for past three months. Performed tasks in time to music, function disrupted when pace too quick. Some control by diverting thoughts, and slowed tempo to improve function, ceased with sleep. Sounds magnified and buzz in head
Dashtipour et al., 2021 [97]^d^	79 m 71 m	6 12	Partial hearing loss RBD symptoms	AH VH, AH, TH, SP	Case 1: Continuous birds chirping softly in musical tone, and faint intermittent music, experienced as intrusive Case 2: Saw daily vague shadows of persons walking alongside in evening. Vivid, exotic animals (e.g. black panther) in living room (no AH description). Felt insects and animals crawling under sheets, presence of people behind. Perceived as intrusive and distressing
Ergun et al., 2009 [98]^d^	80 m	6	No hearing loss; dementia or MDD absent	AH (musical)	Repeatedly heard same two songs (i.e. folk and school march) for months, often in quiet environs, sounded like coming from neighbour’s house (but music persisted with house move). Could reproduce melodies, described as “not his favourite”, but not disturbing, yet no control
Factor and Molho, 2004 [51]^d^	51 f 73 f	21 16	Anxiety, panic, MDD None	AH AH	Case 1: Voice of God with commands to cease medication, eliciting compliance. Voices outside window threatening bodily harm Case 2: Disparaging voices from radio or TV, saying “don’t help her” or “let her rot”. Described as “evil”, threatened to remove medication, and injure, kidnap or poison her
Fenelon et al., 2000 [48]	73.9 ± 7.0, 56% male, 48 71 f 71 m 68 f	12.9 ± 7.5 2 8 18	Dementia (20%); anxiety (28%), delusional disorder (*n* = 1) None Dementia MDD	MSH (VH + AH, AH + SP), VH, AH, SP VH, SP VH, TH, SP MSH (VH + AH), SP	Some VH and AH concurrent, forming “soundtrack of the scene” (15%). AH and SP also often combined. Mean VH 2.2 ± 1.8 years, at least once per day (29%) or per week (39%), or less than weekly (18%), lasting < 5 min (72%), at night (46%) or no dominant time (42%). Persons, animals, or objects, often dynamic (47%). Excellent insight in those without dementia, good insight in those with dementia (64%). Voices (62%), music (14%), or other sounds (48%), such as footsteps. Vivid presence of known relative (15%), guardian angel (2%), rat (2%), or unfamiliar person in room or behind Case 1: Saw two persons in bedroom, followed them to living room and realised not real. Vivid but momentary daily sense of sister lying in bed, at night or upon awakening. Good insight, but repeatedly checked bed. Case 2: Saw “small incorporeal devils with a blurred face”, changing in size, moving rapidly in “sort of haze”. Daily but predominant at night, as entities seemed scared or scattered by light, noise or sudden wave of hand. Became familiar, with intermingling lives, like “living in a fantasy novel” or “parallel world”. Not frightening, with good insight, but admitted to occasional interaction as they “looked so real”. During episode of lumbar pain, felt devils armed with blades butchering back. Presence of entity behind, often turning around to check, but never managing to see its face. Case 3: Saw deceased son (with another person), who said “take care of yourself”. Also presence of unidentified person
Fenelon et al., 2011 [35]	67.0 ± 8.8 (46–84), 62% male, 52	11.5 ± 6.5 (1.5–28)	Dementia excluded	SP	Present for 2.9 ± 4.2 years, at least once per day (31%), or per week (48%), to less than weekly (21%), lasting seconds (79%), minutes (17%) or hours (4%), mostly at night (33%), no predominant time (62%), or upon sleep/wake cycle (6%). Identities were unknown (58%), known (21%), or of deceased (27%). Located behind (27%), to the side (58%), or in other room (12%), when one was indoors (79%), outdoors (6%), or both (15%). Insight was good (77%) or partial/ absent (23%), resulting in checking (79%), with little control (10%). Perceived as static (100%), and described as unpleasant (38%), neutral (54%) or pleasant (8%)
Fenelon et al., 2002 [42]^d^	71 m, 77 m, 64 f, 67 m, 85 f, 77 m, 71 m, 51 m	11.8 ± 6.3 (4–23)	Dementia (*n* = 1); MDD (*n* = 1)	MSH (VH + TH, with some AH or SP)	TH on daily to weekly basis (88%), typically in evening or at night (75%), for six months to 1.5 years (six years maximum). Mostly good insight (75%). Case 1: Painful sensation of cockroaches and spiders nibbling at lower limbs, also saw insects (or dwarfs) biting feet and running away. Felt two “African women” coming up behind, with sensation of their breath and caresses on neck. Saw people moving across walls and sitting alongside. Case 2: Felt wet grubs crawling on neck and left shoulder, saw them on hand after rubbing, and shook hand to get rid of them. Case 3: Skin infested by mites, which also occasionally saw. Case 4: Felt and/or saw rats, shrews and spiders running over lower limbs (mainly feet) or bedsheets during sleep/wake cycle. Kicked out or shouted at them at times. Ceased upon turning on lights, but had occasional sensation that shrew remained in pants when standing. Perceived as unpleasant, but not frightening. Case 5: Presence of husband when lying in bed, accompanied by vivid sensation of bedsheets moving, also heard his breath. Symptoms well-tolerated but fear of stigma. Case 6: Saw ants running on body and floor, also felt ant bites on skin. Caused rubbing of face and body, and requests to moisten skin with water or perfume. Also felt cobwebs on face, “sticky hands”, and lice in hair. Case 7: Sensation of urine running down perineum and legs, lasting several seconds at least once per day, during daytime whilst wearing trousers. Would check for urine, and ask wife to do same. Case 8: Felt struck by “light arrows” and “laser rays” from TV, causing vivid, permanent burning and itching sensations, mainly on forearms and thorax, as evidenced by skin blights or naevi. Heard machine noises and neighbours walking and talking about him. Predominantly nocturnal, but could take place any time of day
Fernandez et al., 1992 [99]	65.0 ± 8.8, na, 30 54 m	12.5 ± 5.7 17	Dementia (50%); MDD (20%)	MSH (VH + AH)	VH + AH (30%), where VH included situations involving humans, such as relatives, children, soldiers, or "strange people” (77%), or wide range of animals (47%), perceived as threatening in some cases (60%). Case 1: Heard war planes bombing central London and saw tanks inside hospital, with people leaving in hurry and strange persons on room walls
Goetz et al., 2011 [38]	66.4 ± 10.6, 52% male, 60	9.0 ± 6.2	Alzheimer’s or delirium excluded	MSH (VH +)	Non-VH progressively emerged over time, with VH plus one other modality onset at 1.5 years, increasing over time, and becoming dominant form at 10 years. Concurrent AH (72%), TH (48%), and OH (21%) in different combinations
Goetz et al., 1982 [100]	na, na, 20	na	No psychosis or other psychiatric disorder; MDD (73%)	MSH (VH + AH), VH, AH	Formed visions and voices, or combined vague sounds with distinct voices, or foggy shadows merging with faces or figures. Visions of humans or animals, and familiar voices or songs. Equal spread of threatening or non-threatening themes, but rarely both together
Gondim Fde et al., 2010 [101]^d^	77 f	1.5	None	AH (musical)	Four musical pieces (two vocal, two unknown) inside head. Constant for up to three days, except when sleeping. Good insight, no control
Gupta et al., 2004 [102]	61.5 ± na, 77% male, 15	4.3 ± na	None	VH, AH, SP	Formed visions of known acquaintances, frightening faces, animals or mythological monsters. Voices convinced that others were plotting against them (27%). Reacted by pressing ear against wall to make out voices (7%). In evening (100%), eliciting distress (67%) and disrupting function (27%). Presence of someone nearby (20%)
Inzelberg et al., 1998 [39]	74.0 ± 9.0, na, 10	6.0 ± 5.0	MDD (*n* = 5), other psychotic features (*n* = 6)	VH, AH	VH and AH not concurrent with unrelated content (no VH description). Repeated voices heard externally, speaking in first- or second-person, with no affective impact. Described as mood incongruent (100%), non-imperative (90%), non-paranoid (90%), and incomprehensible (50%), involving a familiar identity at times (40%). Examples included voice of late husband, commenting on daily activities and giving instructions, which were occasionally obeyed, or voices outside room, possibly over the radio, discussing various topics and eliciting responses. Insight was poor, with voices often perceived as real (90%), and associated with embarrassment (90%)
Jimenez-Jimenez et al., 1997 [103]^d^	66 f	11	None	TH	Somatic feeling of bowels and bladder extruding from distal parts of upper limbs. Compulsively scratched arms, inducing skin erosions
Kataoka et al., 2014 [104]^d^	71 m 65 f 76 m	13.9 7.3 5.9	None	MSH (VH + TH)	Case 1: Saw humans or animals, or described people making bonfires and called police. Insect became enlarged, and tied to or entered body, or felt thin oil put on body. Case 2: Saw humans, and reported snake wound around foot, small animals on abdomen, or insect pricking hip or chest or entering pants or body. Case 3: Daily complaints of lost tooth or thin object entering eyes and associated pains, or “cold thighs” related to passing stools. No VH or AH
Kataoka and Ueno, 2014 [105]^d^	72 f	10	Hearing loss, epilepsy, migraine or schizophrenia excluded	VH, AH (musical at times)	Saw human hands or animals; children talking or rustling noises. Musical hallucinations, involving background music, quiet piano or songs on radio. Not daily, but when occurring, tended to last all day, only ceasing with sleep. Perceived as unpleasant but tolerable. No control, not threatening and no disruption to function. Unrelated to VH or AH
Kataoka and Ueno, 2015 [36]	71.6 ± 6.2, 59% male, 17	4.7 ± 3.2	Dementia, deep brain stimulation or schizophrenia excluded	SP (VH)	Every day (23%), several times per week (47%), once per month (11%), or rarely (17%), lasting seconds (94%) or minutes (5%), in evening (35%), at night (76%), or during day (11%). Indoors (94%) or outdoors (17%), comprising humans (70%), animals (29%), or shadows (11%). Emotional impact included not minding (58%), unpleasant (23%), or terrible (17%), with preserved insight. SP could precede subsequent VH (41%), involving same or different entities
Kesserwani, 2021 [106]^d^	83 f	na	No anxiety or MDD	MSH (VH + TH)	Ants and splinters of wood invading skin, continued vigorous scratching of arms and legs, resulting in excoriation and scabbing. Calm acknowledgement, with minimal distress
Kulick et al., 2018 [46]	67.0 ± 8.0, 54% male, 28	8.5 ± 6.4	Atypical Parkinsonism, or organic psychosis excluded	MSH (OH + GH), VH, AH, OH, TH, GH, SP	Visions of people (14%) or animals (7%), with voices (4%) or music (7%) reported. All OH involved pungent odours, some exclusively foul (14%), involving burning, garbage or other noxious smells. Periodic alterations in odour perception (e.g. food smelling strongly of chemicals) noted (4%). Felt bugs biting or crawling on skin (14%), or cold water dripping on hand (4%). All GH comorbid with OH. Also sensed presence (43%)
Landis and Burkhard, 2008 [52]^d^	57 f 52 f	5 0.5	No epilepsy; hyposmia No smell loss	OH OH	OH preceded PD onset in both cases. Case 1: Brief, repeated and stereotyped episodes, involving strong smells of perfumes, “rainy day” or “wet dog”, lasting seconds to minutes, multiple times a day, with no fixed pattern. Perceived as pleasant. Case 2: Pleasant fragrances, perfumed candles or fruit in evening, in quiet odourless setting, lasting up to 30 min, linked to known identity at times (e.g. mother’s perfume)
Lenka et al., 2018 [107]^d^	57 f	4	None	TH	Prickling from stones, sticks and thorns inside mouth and neck, occasionally falling from mouth (but not visible). Pulling of cheeks, tongue or scalp. Insects and snakes surrounding neck, entering mouth, and biting jaw and tongue, causing breathlessness. Reported great distress and “wanted to die”
Leu-Semenescu et al., 2011 [75]	62.5 ± 11.9, 69% male, 26	7.0 ± 3.6	Narcolepsy; dementia excluded	MSH (VH + AH, AH + SP), VH, AH, TH, SP	Mostly recurrent (92%), during sleep/wake cycle (46%), with poor insight (24%). VH and AH concurrent, woman lying in bed between self and husband, saying “I’ll replace the drugs with a poison”. Resulted in sleeplessness and refusals to eat or take medication. AH and SP concurrent, with unknown presence behind when walking at river’s edge in evening. Increased pace and started running, but could hear footsteps following, eliciting fear. TH and SP concurrent comprising smooth touch of hand on shoulder, or pet or insect (31%). Visions involved people or animals moving, or woman in white dress climbing on bell tower of church. Verbal (e.g. threat words) or non-verbal (e.g. footsteps) sounds. Human or animal (e.g. fur of cat or dog lying at feet) touch. Presence of known person, such as relative, or pet sitting quietly behind (58%)
McAuley and Gregory, 2012 [78]^d^	71 m 70 f 77 f 74 m	8 14 10 6	No dementia or smell loss for all cases	VH, OH VH, OH OH VH, AH, OH	Case 1: Saw rats crawling under carpet. Smelt unpleasant odours from bedsheets at night, lasting minutes to hours, even away from bed. Frequently bought new sheets, and developed obsessive thoughts. Case 2: Saw insects and vague shadows. Nocturnal dog faeces smells, lasting an hour and disrupting sleep. Case 3: Early morning smells of burning ash for several minutes. Case 4: Saw spiders crawling on shirt, and “tinnitus” involving factory noise. Nocturnal bonfire odours for several minutes, perceived as unpleasant (nb. two other unidentified cases reported boot polish and perfume odours)
Meco and Bernardi, 2007 [108]^d^	60 m	8	Anxiety, MDD	MSH (VH + AH)	Police officers outside house, accompanied by sirens and threatening voices. Prompted refusals to leave house, affecting mood and sleep. Fearful of strangers in house
Mellers et al., 1995 [59]	64.9 ± 10.1, 63% male, 14	12.6 ± 5.7	Dementia excluded	VH, AH, TH	Images of groups of people (57%), animals or insects (36%), inanimate objects (21%), simple forms (14%), or Lilliputian hallucinations (14%). Third- (21%) or second-person (7%) voices, or other sounds (7%). Also being touched (21%)
Mittal and Giron, 2010 [109]^d^	60 m	10	Hearing loss; compulsive gambling	MSH (VH + AH, musical at times)	Saw deceased uncle fishing alongside, saying “it’s not going to work”. Simple images of silhouettes, shadows or animal-like shapes. Complex visions included woman sitting alongside in car, two well-dressed mice running, chimpanzee drinking milk next to lunch table, or Elvis Presley in white without guitar. Musical hallucinations described as incomprehensible, non-specific symphony, like constant melody of chimes. Perceived as non-threatening, with good insight
Moskovitz et al., 1978 [110]	na, 56% male, 31	na (0.5–20)	Severe dementia and psychosis history excluded	MSH (VH + AH), TH	Predominantly VH (secondary AH at times), mostly nocturnal and recurrent, non-threatening (73%), and stereotyped. Images of significant individuals and experiences from past, blending with dreams comprising comparable themes (no TH description)
Muralidharan et al., 2011 [111]^d^	72 m	7	Insomnia	VH, AH	Voices in third-person over past two months, with no direct impact on mood, but reported distress (no VH description)
Nagata et al., 2013 [112]^d^	48 f	7	None	AH	Voices began as hearing strangers speak when alone. Gradually worsened, prompting daily comments, “a stranger came here again”, or asking them to “go away”
Nishioka et al., 2014 [113]^d^	54 f 65 m	10 9	Anxiety MDD	TH MSH (VH + AH)	Case 1: Felt ice blocks covering surface of entire body Case 2: Two monsters blaming him, and human faces on wall laughing at him
Onofrj et al., 2000 [114]^d^	81 m	6	None	MSH (VH + AH + TH)	Vivid daytime and nocturnal visions of intruders entering bedroom from wardrobe and laying on bed. Heard them talking and felt their bodies on bed. Perceived as annoying and uncomfortable, but non-threatening
Pagonabarraga et al., 2016 [65]	68.8 ± 10.0, 56% male, 21	19.5 ± 15.0	PD drug-naïve; no major cognitive impairment or major psychiatric disorders	VH, AH, OH, SP	More than once per week (57%), with preserved insight. Transient images of faces through window, or insects crawling on table (10%). Heard person knocking on door, and footsteps inside apartment (5%). Smelt burning wood and plastic (10%). Presence of known person (e.g. partner, sibling, carer, deceased spouse) in close proximity behind shoulder, whilst sitting or engrossed in household tasks, reacted by turning head to check. At follow-up, hallucinations remained stable (52%), deteriorated (38%), or remitted (10%)
Paleacu et al., 2005 [83]	76.0 ± 7.0, 30% male, 76	7.8 ± 5.2	DLB excluded	MSH (VH + AH), VH, TH	VH comprising images of humans, often family members, some deceased, of regular size and colour, and silent, as well as animals (4%), or tree branch (1%). Background voices (8%), described as mood congruent, non-paranoid, and incomprehensible, but rarely imperative. All AH comorbid with VH. Felt person touching toes (1%)
Papapetropoulos et al., 2008 [84]	55.4 ± 12.5, 68% male, 31	9.5 ± 5.6	No drug-induced delirium or hallucinations related to another medical illness	MSH (VH + AH, VH + TH), VH, AH, OH, TH	At least once per week (56%), only a few times (16%), or less than weekly but ongoing (28%), with sudden (68%) or gradual (13%) onset. Instantaneous (32%), lasting < 10 s (58%), or ≥ 10 s (3%), at night (29%), or any time of day (58%), and familiar (39%). Good (36%), mixed (26%), or poor (32%) insight. Perceived as friendly/indifferent (42%), or distressing (58%), ranging from mild (19%,) moderate (16%) to severe (23%). Single (65%) or multiple (35%) modalities, with a proportion non-visual (23%). VH and AH concurrent (16%), including human silhouettes or people running, alongside voices and dogs barking. VH and TH concurrent, seeing shadow and feeling one sitting on chest. Visions typically familiar or unfamiliar persons, solid, real-sized, coloured, and perceived as non-threatening, appearing momentarily, possibly transparent (6%), or distorted in size (10%). Other instances included neighbour’s children, unfamiliar male faces, fragmented bodies, little bears, spider webs and a luminous ball. Voices (of parents) and conversations, people arguing, “psst” sounds reminiscent of bird. Odours of cleaning products, gas or medicine
Parsa and Bastani, 1998 [115]^d^	74 f	6	None	VH, AH	Saw animals running in apartment, and heard deceased husband whistling at night
Perez-Perez et al., 2016 [62]^d^	75 m, 92 f, 83 m, 79 m, 76 m, 85 f, 80 f	13.6 ± 10.6 (6–37)	Sensory or neurological disturbances excluded	MSH (VH + TH, some SP)	Unpleasant vision and sensation of threads (i.e. long strings or yarn) emerging from hands, prompting attempts at removal (e.g. gesturing to wind into a ball to offload), due to annoyance caused. Frequency fluctuated, but recurrent in those with dementia, likely in evening, related to sleepiness, and accompanied by varying insight. Case 1: Strips of paper or threads of wool growing out of fingers and palm. Also saw fleeting images of shadows. Case 2: Silk threads (or cobwebs at times) coming out of fingertips, or familiar person patting shoulder, as well as SP. Case 3: Yarn rolled around index and middle finger when seated with hands on lap, would try to unravel. Case 4: Daily playing with threads in hands, changing from one hand to other, mostly in evening when sleepy, accompanied by frequent VH. Case 5: Played with threads and hairs around fingers, also saw deceased mother. Case 6: Strings coming out of hands, also saw children playing around house, who sometimes touched her and called her “grandma”. Case 7: Threads on fingers, causing scratching of hands during attempted removal, also visions of unknown persons in house, perceived as threatening
Potheegadoo et al., 2022 [116]^d^	73 m 79 f	14 7	Dementia excluded in both cases	SP (TH) SP (AH)	Case 1: Presence behind on right peering over shoulder when walking. Perceived person wants to overtake as they walk faster, and when two steps behind, moves aside to let them pass (no TH description, except experienced when still). Case 2: A positive and negative presence, occurring independently several times a month, when walking. Positive guardian angel present for few seconds, but negative impression lasted longer, next to shoulder on right, feeling like they are catching up, and letting them pass (no AH description)
Rai et al., 2015 [85]	63.6 ± 8.0, 27% male, 30	8.6 ± 3.4	MDD (44%), psychosis (24%) or anxiety (36%)	MSH (VH + AH), TH	VH and AH concurrent (27%), involving living or deceased relatives conversing with oneself (10%) or offering a running commentary (20%). Saw animals, monsters or shadows. Felt insects creeping over skin, followed by unusual positive sensation over trunk and limbs
Rana et al., 2013 [47]	76.0 ± 8.9, 44% male, 34	na	Dementia (41%); history of psychotic disorders, or drug-induced psychosis excluded	MSH (VH + TH), VH, AH	VH and TH concurrent, such as insects on skin (3%), person in bed (3%), or arms growing longer (3%). Saw familiar (9%) or unfamiliar (77%) people in or outside home, animals or insects (44%), or objects (15%). Most reported one type of VH (68%), but up to five types endorsed. Perceived as bothersome (59%), threatening (15%), or needing treatment (59%). Heard voices of deceased family members or household noises (6%). Reported being bothered by running commentary (3%). Some reduced insight (15%)
Reckner et al., 2020 [49]	67.7 ± 5.2, 78% male, 18	9.5 ± 6.2	Also further DLB patients (*n* = 7) included	SP	Human (88%), animals (29%), or shadows (18%), involving one type (88%), but two or more types (12%) at times. At least once per day (24%), or per week (29%) to less than weekly (47%), mostly lasting < 1 s (35%), < 10 s (29%), or ≥ 10 s (35%). Located directly behind (82%), or further away (18%), often indoors (53%) in kitchen or living room, but could be outdoors (12%), or both (12%), at different times of day, largely with neutral valence (67%). Patients with good insight (39%) described presence as not real (100%) and unfamiliar (86%), whereas those with reduced insight noted deceased or living identity (70%), and took corresponding action/interaction (e.g. reported feeling touch), with greater behavioural impact, in terms of checking (70%), emotional distress (50%), and perceived control (60%). Attributed to physical causes (94%), with those with retained insight ascribing benign meaning (100%), but those with reduced insight perceiving as negative (50%)
Roberts et al., 1989 [117]^d^	64 f	10	MDD	VH, AH	Persistently saw several people, including family members, wearing white sheets and hiding behind trees in yard at night. Heard voice of deceased husband calling to her
Solla et al., 2021 [34]	73.9 ± 8.1, 25% male, 16	3.9 ± 3.5	Dementia and psychiatric disorders excluded	MSH (OH + GH), OH	Odours unpleasant (19%), such as garbage, rotten eggs or other noxious smells, or pleasant (81%), like flowers or fruit. All GH comorbid with OH (25%)
Ting et al., 2019 [54]^d^	60 + m	6	MDD	MSH (AH + SP)	Presence of person at bedside despite being alone, and hearing their breathing at times, lasted for few seconds, often upon awakening
Tousi and Frankel, 2004 [118]^d^	78 f	15	MDD	VH, OH	Described seeing “little people”. Smelt burning grass for hours at any time of day. Dry throat from smoke, perceived as irritating
Voon and Lang, 2004 [119]^d^	57 m	7	Panic, MDD and psychosis	MSH (VH + AH)	Persistent nocturnal experiences of people coming out of ceiling and speaking with him, also saw pipes coming out of wife. Several hours nightly, not related to sleep/wake cycle. Nocturnal images of smoke upon awakening, lasting 5 min weekly, perceived as not distressing
Wand, 2012 [120]^d^	80 m	1	MDD; cognitive impairment and other psychiatric history absent	AH	Heard voice calling name, and responded by checking door. Became scared when realised no one was there. No hallucinations in any other modalities
Whitehead et al., 2008 [55]	75.1 ± 7.5, 74% male, 27	6.2 ± 5.2	Moderate to severe dementia, suspected DBL, or other neurological diseases excluded	MSH (VH + AH, VH + SP, TH + SP), VH, AH, OH, TH, SP	At night (33%), during day (26%) or both (41%), with good (44%) or poor (56%) insight. VH and AH concurrent, involving “burglar” hiding behind wardrobe, having sex with girlfriend. VH and SP, where children playing in house. TH and SP concurrent, with deceased husband sitting at end of bed. Visions of figures and animals, such as threatening faces or figures in bedroom, or man in bell boy uniform with unpleasant demeanour and “devilish” identity. Workmen digging holes in floor at night, or miniature “flower people” mowing lawn. “Lenin’s embalmed corpse” in bed, deceased husband sitting on chair, deceased cat on chest of drawers, or angel in pointed hat in living room. Also “dancing girls”, tiger in garden, bird in house, or “fire and flames”. Voices included person talking in next room or elsewhere in flat, loud voices outside window, or in the night. Burning, cigarette smoke or rotting fish odours. Sensation of deceased dog jumping on bed and lying at feet, or creatures in bed. Bugs crawling or feather moving across skin. Presence of sister lying in bed, one sitting in armchair in evening, friends in living room, woman walking alongside upstairs each morning, or children playing
Wood et al., 2015 [37]	62.1 ± 8.1, 46% male, 208	7.5 ± 5.0	DBL, dementia or neurogenerative disorders excluded	MSH (VH + SP), SP	When comorbid with VH (17%), concurrent (53%), before (25%) or after (22%). Possible AH (6%) or TH (11%). Related to familiar (34%) or unfamiliar (66%) people (71%), animals (20%) or objects (9%), often described as “feeling of movement passing” (82%) or “felt or imagined presence” (72%). Not stereotyped (73%), or associated with particular time of day (74%), appearing to the side (90%), or left or right (47%), and perceived as emotionally neutral (67–81%)
Yoshida et al., 2009 [121]	63.2 ± 7.8, 38% male, 13	13.8 ± 5.1	None	VH, AH, SP	Images of dead pet bird on roof, deceased brothers standing by gate, falling needles, bugs on duvet, face in air, animals or flowers, or being attacked by people. Voice of deceased mother, karaoke sounds, or people calling, frightening at times. Presence of moving black shadow, people or figures walking around, in front or behind
Zhong et al., 2021 [90]	65.7 ± 8.1, 53% male, 28	6.1 ± 4.3	DBL, major psychiatric disorders or use of antipsychotics excluded	SP	Familiar (14%) or unfamiliar (79%) persons, or objects (4%), at least daily (18%), less than daily (71%) or weekly (11%), lasting seconds (82%) to minutes (18%), in the day (54%), at night (25%) or both (21%), with sudden onset (82%). In dim (64%) or bright (25%) lights, mostly indoors (86%), regular-sized (93%), blurry (93%), and in black-and-white (86%). Stereotyped (43%) or miniaturised (7%)
Zhu et al., 2017 [91]	67.8 ± 8.8, 68.1% male, 72	7.7 ± 5.2	Atypical Parkinsonism or dementia excluded	MSH (VH + AH), VH	AH accompanied by VH (21%), characterised by repetitive human voice. Complex visual images of mundane content, involving familiar (44%), unfamiliar (47%) or deceased (17%) persons (61%), animals (31%) or inanimate objects (25%). At least daily (28%), less than daily (36%) or weekly (36%), lasting seconds (63%) to minutes (33%), in dim (53%) or dark (24%) surrounds, with sudden onset (76%). Stereotyped (49%), somewhat blurry (63%) and often dynamic (85%), in black-and-white (36%), single- (33%) or multi-coloured (31%), regular-sized (56%), miniaturised (28%) or magnified (17%). Accompanied by heightened reality (60%), and good insight (78%)

*PD* Parkinson’s disease; *m* male; *f* female; *na* not available; *MSH* multisensory hallucinations; *VH* visual hallucinations; *AH* auditory hallucinations; *OH* olfactory hallucinations; *TH* tactile hallucinations; *GH* gustatory hallucinations; *SP* sensed presence, also referred to as “feeling of presence” or “extracampine hallucinations”; *RBD* rapid eye movement (REM) sleep behaviour disorder; *MDD* major depressive disorder; *DLB* dementia with Lewy bodies

^a^Maximum range provided in brackets (where available)

^b^For group design studies only

^c^Numbers of participants endorsing each phenomenological facet expressed as percentages (where available) to facilitate comparisons across studies (as *n* differed); where these do not total to 100%, this either reflects missing data, or items where endorsement of more than one response option was permitted

^d^Denoting case studies; group design studies if not otherwise specified, except for Fenelon et al., 2000 [38], which constituted both a case and group design study

### Significant clinical and cognitive correlates of modality-specific hallucinations in PD

Select clinical and cognitive correlates of modality-specific hallucinations in PD are presented in Table 3, where 2008 patients across 16 studies were included. Subgroups for comparisons were those with auditory, olfactory (or with or without visual) hallucinations or sensed presence (assessed against control groups without hallucinations or psychosis at times). Broadly, it can be concluded that participants with any modality-specific hallucinations fared worse across all clinical and cognitive variables, when significant findings were established (though non-significant findings were also recorded). The picture was less clear when considering correlates from a modality-specific lens; PD patients with olfactory hallucinations had significantly more severe PD (1/2 studies) and poorer cognition (1/2 studies). Similarly, those with sensed presence had significantly increased length of illness (1/1 studies), more severe PD (2/2 studies), and poorer cognition overall or in select domains (2/2 studies). The two studies involving auditory hallucinations had too small subgroup numbers to form meaningful conclusions.

**Table 3:** Select clinical and cognitive correlates of modality-specific hallucinations in Parkinson’s disease (*n* = 12 studies)

Author, year	Participant subgroup/numbers	Clinical correlates						Cognitive correlates
		Age (years)	Age of PD diagnosis	Length of illness (years)	PD severity (HY or MDS-UPDRS)	Depression	Dementia	
Barrett et al., 2017 [67]	PD_vh_ = 28 vs PD_psy-_ = 68	ns	ns	ns	UPDRSII: PD_vh_ > PD_psy-_, *p* = 0.001 UPDRSIII: PD_vh_ > PD_psy-_, *p* = 0.019	BDI-II: PD_vh_ > PD_psy-_, *p* = 0.016	–	ns for MoCA, Trails A + B, HVLT subtests, COWA, matrix reasoning and JLO
Fenelon et al., 2000 [48]	PD_vh_ = 48 (or PD_hall+_ = 86)^a^ vs PD_hall-_ = 130	PD_vh_ > PD_hall-_, *p* = 0.001	ns	PD_vh_ > PD_hall-_, *p* < 0.001	HY: PD_vh_ > PD_hall-_, *p* < 0.001 UPDRSI: PD_vh+_ > PD_hall-_, *p* < 0.001 UPDRSII: PD_vh+_ > PD_hall-_, *p* < 0.001	CES-D: PD_vh_ > PD_hall-_, *p* = 0.007	PD_vh_ > PD_hall-_, *p* < 0.001	MMP: PD_vh_ < PD_hall-_, *p* < 0 .001
Fenelon et al., 2011 [35]	PD_sp+_ = 38 vs PD_sp-_ = 78 (or PD_vh+sp_)	ns	–	PD_sp+_ > PD_sp-_, *p* = 0.006; PD_vh+sp_ > PD_sp+_, *p* = 0.04	HY: PD_sp+_ > PD_sp-_, *p* < 0.001	–	–	MMP: PD_vh+sp_ > PD_sp+_, *p* = 0.002
Holroyd et al., 2001 [71]	PD_vh_ = 26 vs PD_hall-_ = 72	–	–	–	UPDRSI: PD_vh_ > PD_hall-_, *p* = 0.008	GDS: PD_vh_ > PD_hall-_, *p* = 0.014	–	TICS: PD_vh_ > PD_hall-_, *p* = 0.002
Inzelberg et al., 1998 [39]	PD_vh_ = 35 vs PD_vh+ah_ = 10 vs PD_hall-_ = 76	ns	–	ns	–	–	PD_vh_ > PD_hall-_, *p* < 0.001	SMT: PD_vh_ > PD_hall-_, *p* < 0.001
Katzen et al., 2010 [56]	PD_vh_ = 47 vs PD_hall-_ = 105 (also PD_vhonly_ vs PD_vh+_)^b^	ns	ns	PD_vh_ > PD_hall-_, *p* = 0.05	HY: ns	BDI-II: PD_vh_ > PD_hall-_, *p* = 0.005	–	JLO: PD_vh_ < PD_hall-_, *p* = 0.02; PASAT (5 s): PD_vh_ < PD_hall-_, *p* = 0.04; mWCST: PD_vh_ > PD_hall-_, *p* = 0.05–0.01; ns for MMSE, BNT, COWA, BVRT, HVOT, GEFT, CVLT, Trials A + B, SDMT
Marques et al., 2021 [122]	PD_vh_ = 28 vs PD_vi_ = 26	ns	–	ns	HY: PD_vh_ > PD_vi_, *p* = 0.02 UPDRSI: PD_vh_ > PD_vi_, *p* < 0.001 UPDRSII: PD_vh_ > PD_vi_, *p* = 0.002 UPDRSIII, UPDRSIV: ns	–	–	MoCA: PD_vh_ < PD_vi_, *p* = 0.04; MDRS Initiation: PD_vh_ < PD_vi_, *p* = 0.04, ROCF: PD_vh_ < PD_vi_, *p* = 0.04; ns for other MDRS subtests
Matsui et al., 2007 [41]	PD_ah+_ = 4 vs PD_ah-_ = 77	ns	–	ns	HY, UPDRSII: ns	ns	–	MMSE: ns
Reckner et al., 2020 [49]	PD_sp+_ = 18 vs PD_sp-_ = 25	ns	–	ns	–	ns	–	Visual processing, executive function: PD_sp+_ < PD_sp-_, *p* < 0.05; processing speed: PD_sp+_ < PD_sp-_, *p* < 0.001; ns for memory and language
Solla et al., 2021 [34]	PD_oh+_ = 16 vs PD_oh-_ = 125	ns	–	ns	UPDRSIII: PD_oh+_ > PD_oh-_, *p* < 0.001	–	–	MoCA: PD_oh+_ < PD_oh-_, *p* = 0.035
Wood et al., 2015 [37]	PD_sp+_ = 208 vs PD_sp-_ = 204	ns	–	ns	HY: PD_sp+_ > PD_sp-_, *p* = 0.002	–	–	–
Zhu et al., 2017 [91]	PD_vh_ = 72 vs PD_hall-_ = 299	PD_vh_ > PD_hall-_, *p* = 0.01	ns	PD_vh_ > PD_hall-_, *p* = 001	HY: PD_vh_ > PD_hall-_, *p* = 0.001 UPDRSIII: PD_vh_ > PD_hall-_, *p* < 0.001	ns	–	MoCA: PD_vh_ < PD_hall-_, *p* = 0.001; attention: PD_vh_ < PD_hall-_, *p* < 0.001; orientation: PD_vh_ < PD_hall-_, *p* = 0.006; ns for executive/ visuospatial, abstraction, delayed memory, language, and naming

*PD* Parkinson’s disease; *PD*_*hall*+_ PD with hallucinations; *PD*_*hall-*_ PD without hallucinations; *PD*_*vh*_ PD with visual hallucinations; *PD*_*psy-*_ PD without psychosis; *PD*_*sp*+_ PD with sensed presence; *PD*_*sp-*_ PD without sensed presence; *PD*_*vh*+*sp*_ PD with visual hallucinations and sensed presence; *PD*_*vh*+*ah*_ PD with visual and auditory hallucinations; *PD*_*vhonly*_ PD with visual hallucinations only; *PD*_*vh*+_ PD with visual hallucinations, plus hallucinations in one or more sensory modalities; *PD*_*vi*_ PD with visual illusions; *PD*_*ah*+_ PD with auditory hallucinations; *PD*_*ah-*_ PD without auditory hallucinations; *PD*_*oh*+_ PD with olfactory hallucinations; *PD*_*oh-*_ PD without olfactory hallucinations; *HY* Hoehn-Yahr scale; *UPRDRS* Movement Disorders Society Unified Parkinson Disease Rating Scale; *ns* not significant; *MMSE* Mini Mental State Examination; *BDI-II* Beck Depression Inventory; *MoCA* Montreal Cognitive Assessment; *HVLT* Hopkins Verbal Learning Test; *COWA* Controlled Oral Word Association; *JLO* Benton Judgment of Line Orientation; *CES-D* Centre for Epidemiological Studies-Depression Scale; *MMP* Mini Mental Parkinson; *GDS* Geriatric Depression Scale; *TICS* Telephone Interview for Cognitive Status; *SMT* Short Mental Test; *PASAT* Paced auditory serial addition task; *mWCST* modified Wisconsin Card Sort Test; *BNT* Boston Naming Test; *BVRT* Benton Visual Retention Test; *HVOT* Hooper Visual Orientation Test; *GEFT* Ghent Embedded Figures test; *CVLT* California Oral Word Association Test; *SDMT* Symbol Digit Modalities Test; *MDRS* Mattis Dementia Rating Scale; *ROCF* Rey-Osterrieth Complex Figure

^a^Same pattern of significant group findings when patients with hallucinations in any modality were employed (*p* values not available)

^b^No significant clinical or cognitive differences were identified between these groups (nb. Bannier et al., 2012 reported no significant differences between those with and without olfactory hallucinations in terms of age, sex, age of PD onset, duration and severity of PD illness, medication use, MMSE cognition, mood states as well as odour detection and identification; not included in table, as no statistics provided)

Significant associations or risk factors for modality-specific hallucinations in PD are shown in Table 4. Significant associations were identified between increased modalities of hallucinations and poorer overall and domain-specific cognition, as well as between olfactory hallucinations and female sex, presence of visual/auditory hallucinations and PD severity. Significant predictors of olfactory hallucinations were female sex and presence of visual or auditory hallucinations in one study [34], whereas significant predictors of sensed presence were PD severity, poorer cognition, presence of visual hallucinations or illusions, and increased levodopa equivalent (or other PD medication) dose across three studies [35–37]. Notably, Goetz et al. [38] demonstrated that length of illness at baseline, time elapsed, and first onset of visual and non-visual hallucinations predicted future increased occurrence of visual and non-visual hallucinations.

**Table 4:** Significant associations or predictors for modality-specific hallucinations in Parkinson’s disease (*n* = 10 studies)

Author, year	Participant age (years), sex (% male), numbers	Significant associations or predictors	Covariates (if any)	Statistics (*r* or OR, CI, *p*)	Variable of interest
Barrett et al., 2017 [67]	67.3 ± 10.6, 59, 101	Dopamine agonist PD severity (UPDRSII) RBD	–	OR = 5.0, *p* = 0.007 OR = 1.1, *p* = 0.010 OR = 4.8, *p* = 0.012	Visual hallucinations
Factor et al., 2014 [70]	62.8 ± 9.3, 68, 25	Cognition (MMSE, attention, language, memory, visuospatial abilities)	–	*r* = −0.53**, *r* = −0.44*, *r* = −0.55**, *r* = −0.41*, *r* = −0.49*	Hallucination modality
Fenelon et al., 2000 [48]	69.2 ± 8.9, 57, 178	Cognition (MMP < 24) Length of illness (> 8 years) Daytime somnolence	–	OR = 10.3, CI = 4.3–25.1, *p* < 0.001 OR = 3.1, CI = 1.3–7.6, *p* = 0.01 OR = 3.5, CI = 1.4–5.0, *p* = 0.006	Visual hallucinations
Fenelon et al., 2011 [35]	67.1 ± 9.9, 65, 116	Visual hallucinations Visual illusions LED (> 750 mg per day)	–	OR = 1.7, CI = 1.1–2.8, *p* = 0.029 OR = 4.6, CI = 1.6–12.8, *p* = 0.004 OR = 4.5, CI = 1.2–16.4, *p* = 0.023	Sensed presence
Goetz et al., 2011 [38]	66.4 ± 10.6, 52, 24–60	Length of illness (at baseline) Time elapsed (10 years) First visual and non-visual hallucinations	PD medications	OR = 1.1, CI = 1.0–1.2, *p* = 0.01 OR = 1.2, CI = 1.1–1.3, *p* < 0.001 OR = 3.7, CI = 1.1–11.9, *p* = 0.03	Visual and non-visual hallucinations
Kataoka and Ueno, 2015 [36]	na, na, 58	PD severity (HY, UPDRS) cognition (MMSE) PD medications	–	OR = 0.1–1.3, CI = 0–1.6, *p* = 0.039–0.042 OR = 0.5, CI = 0.3–0.9, *p* = 0.025 OR = 70.8, CI = 2.3–2150.4, *p* = 0.014	Sensed presence
Rana et al., 2013 [47]	74.1 ± 10.3, 57, 323	Sex (female) Dementia PD severity (HY) Unfamiliar people Bothersome Other animate objects	– Bothersome Treatment-seeking Treatment-seeking	OR = 2.4, CI = 1.1–5.3, *p* = 0.031 OR = 4.7, CI = 2.0–11.1, *p* = 0.001 OR = 7.6–9.1, CI = 2.4–33.8, *p* = 0.001 OR = 24.9, CI = 1.4–439.8, *p* = 0.028 OR = 27.1, CI = 2.7–276.7, *p* = 0.005 OR = 6.9, CI = 1.2–40.2, *p* = 0.032	Visual hallucinations Visual hallucination content
Solla et al., 2021 [34]	70.2 ± 9.4, 60, 141	Sex (female), visual or auditory hallucinations, PD severity (UPDRSIII) Sex (female) Visual or auditory hallucinations	– –	*r* = 0.26**, *r* = 0.36**, *r* = 0.20* OR = 3.9, na, *p* = 0.034 OR = 4.7, na, *p* = 0.017	Olfactory hallucinations
Wood et al., 2015 [37]	61.9 ± 8.2, 52, 414	Presence of visual hallucinations	–	OR = 7.1, 3.4–14.9, *p* < 0.001	Sensed presence
Zhu et al., 2017 [91]	65.3 ± 9.1, 67, 371	Length of illness Cognition (MoCA attention/orientation) Dopamine agonist Non-motor symptoms (NMSQ)	–	OR = 1.1, 1.1–1.2, *p* = 0.011 OR = 0.4–0.5, 0.2–0.8, *p* = 0.001–0.015 OR = 2.0, 1.1–3.6, *p* = 0.029 OR = 1.5–1.6, 1.2–2.2, *p* = 0.001–0.005	Visual hallucinations

*OR* odds ratio; *CI* 95% confidence interval; *na* not available; *PD* Parkinson’s disease; *HY* Hoehn-Yahr scale; *UPRDRS* Movement Disorders Society Unified Parkinson Disease Rating Scale; *RBD* rapid eye movement (REM) sleep behaviour disorder; *MMSE* Mini Mental State Examination; *MMP* Mini Mental Parkinson; *LED* levodopa equivalent dose; *MoCA* Montreal Cognitive Assessment; *NMSQ* Non-motor Symptoms Questionnaire

*p < 0.05, **p < 0.01

### Risk of bias assessment

For the 30 case studies rated on the JBI-CAC, 90% were *excellent*, and 10% were *good*. For the 62 empirical studies rated on the NOQAS, 19.4% were *excellent*, 38.7% were *good*, 29% were *fair*, and 12.9% were *poor* (see Tables E and F in Supplementary materials). A significant fraction of adverse ratings for case and group design studies were due to lack of valid hallucination assessments, with a proportion of group design studies also marked down owing to lack of a control group for comparison (as prioritised in the tool employed).

## Discussion

This systematic review aimed to examine the prevalence, phenomenological characteristics as well as major clinical and cognitive correlates of non-visual or multisensory hallucinations in PD, by collating findings from existing studies in the field. For our first research question regarding prevalence, rates for auditory (and visual) hallucinations as well as sensed presence were largely in line with figures cited in the literature [10–13]. There is no doubt that visual hallucinations (up to 96.9%), alongside sensed presence (up to 73.3%), remain the most typical sensory mode in PD. Most reviewed studies were of prospective, cross-sectional design, with those specifying prevalence as their predominant aim likely offering more accurate numbers. Though prevalence estimates for olfactory, somatic-tactile and gustatory hallucinations were of lesser magnitude (than visual hallucinations), sizeable figures (15–22.5%) were still recorded, signifying that these phenomena clearly affect a notable proportion of PD patients. Yet these wide-ranging numbers were also subject to methodological variations in terms of patient selection, evaluation methods, as well as possible divergence in symptoms assessed, thereby precluding conclusive prevalence rates being endorsed.

For our second research question on phenomenology, anecdotal descriptions of non-visual and multisensory hallucinations in PD demonstrated variability in individual experience, with little consistency across any of the physical, cognitive or emotional characteristics (see Table 2). In other words, *frequency, duration, intensity, clarity, localisation, time of occurrence, personification, controllability, conviction, interaction, beliefs regarding origin, content, valence, distress* and *functional impact* varied within and between individuals, depending on the specific hallucination episode and/or modality under examination. Similarly, whilst visual involvement in multisensory hallucinations was likely, non-visual symptoms, whether in single or multiple modalities, were also observed. These hallucinations were typically described with graphic detail, with PD patients fully immersed in their experiences. Coupled with the fact that these events were often not spontaneously divulged [40], this raises the speculation of whether non-visual hallucinations in PD may actually be more clinically significant than previously postulated. Involvement of greater number of sensory modalities in multisensory hallucinations also begets the question of whether there could be an incremental adverse impact on PD patients who have these experiences (see Sect. "Clinical implications and recommendations").

For our third research question, we focused on elucidating significant clinical and cognitive correlates of modality-specific hallucinations in PD. At the outset, we need to qualify that findings relating to the visual modality are incomplete. This is because our aim and search strategy focused on non-visual and multisensory hallucinations (with results for visual hallucinations presented alongside, only when they were concurrently analysed). Notably, our review demonstrated a clear lack of relevant studies in non-visual domains. Only a handful of studies were uncovered in auditory and olfactory hallucinations as well as sensed presence (see Tables 3 and 4), with the remaining focused on the visual domain (see Tables C and D). Nonetheless, we largely corroborated that PD patients with hallucinations (in any modality) tended to fare significantly worse in terms of increased length of illness, greater PD severity, presence of major depressive disorder and/or dementia as well as poorer cognition overall and in specific domains [18]. Other than depression, these variables have been verified as significant risk factors for developing hallucinations [10]. Furthermore, we identified added factors of interest to modality-specific hallucinations, where visual hallucinations were identified as a significant risk factor for sensed presence, and female sex and visual or auditory hallucinations were identified as significant risk factors for olfactory hallucinations. Yet caution must be exercised when focusing on findings relating to non-visual hallucinations owing to: (i) limited number of studies, (ii) small subgroup sizes [40, 41], and (iii) comorbid visual hallucinations in those subgroups [34, 41]. Further replicative efforts are thus essential.

Finally, our risk of bias assessment revealed that case studies were generally of high quality, whereas empirical studies were of mixed quality, meaning we need to be mindful of these limitations when drawing interpretations and subsequent conclusions from the latter body of work.

### Clinical implications and recommendations

There are several clinical implications and recommendations we can draw from our findings. First, non-visual and multisensory hallucinations in PD are likely of clinical significance. Empirical evidence supporting this statement comes from prevalence figures noted, coupled with vivid phenomenological information, substantially contributing to patient distress in some cases. To increase focus in this area, clinicians and researchers first need to specifically ask about patient experiences of non-visual and multisensory symptoms during routine assessments. Some PD patients are unlikely to volunteer these symptoms because of embarrassment or fear of stigma in trying to avert a further psychiatric diagnosis [39, 42]. Being transparent and matter-of-fact about asking these questions should somewhat allay these concerns. Having a well-validated assessment tool will also be helpful in guiding conversations. Though the Movement Disorders Society Unified Parkinson Disease Rating Scale (UPDRS) remains the gold-standard measure, hallucinations (not modality-specific) and delusions are jointly addressed within a single item (nb. for this reason, PD studies that assessed hallucinations with this measure only were not able to be included within the current review). A brief accompanying hallucinations screen, such as the Psycho-Sensory Hallucinations Scale [43, 44], will circumvent this issue. More comprehensive investigations can alternatively employ the Questionnaire for Psychotic Experiences [45]. Related to this, sensed presence (included within this review), alongside passage hallucinations and visual illusions, has often been termed a ‘minor’ hallucination [46], implicitly deserving of lesser concern. This in turn suggests that hallucinatory experiences in other forms are ‘major’ in nature. Being cautious about such terminology may aid in managing patients’ views and expectations of these symptoms, noting that ‘minor’ hallucinations are often (though not always) comorbid with non-visual (and visual) hallucinations in PD.

Second, a factor that does motivate self-report of hallucinations in PD is when a patient is bothered by these experiences [47]. Many patients with symptoms established as characteristic in clinical presentations for PD, including visual hallucinations and sensed presence, exhibit good insight and minimal distress, despite the somewhat anomalous nature of these events [49, 50]. We can therefore speculate that a lack of patient awareness of non-visual hallucinatory symptoms in the disorder may contribute to feelings of fear and anxiety. Moreover, involvement of increasing number of modalities in multisensory hallucinations may further elevate patient distress (and possibly reduce insight), owing to enhanced veracity of the experience given multiple sensory inputs, especially if these coincide temporally. Supportive therapies that may be introduced to patients and/or their carers include psychoeducation, cognitive-behaviour techniques (if suitable), and where hearing loss may be a factor, the use of hearing aids [50]. It is hoped that simply asking about these experiences, and putting basic management strategies in place may be of benefit in the first instance.

Third, some authors have suggested that non-visual hallucinations tend to emerge in older age [14] or later-stage PD [13], but empirical support for this is still tentative. The most convincing evidence to date stems from a 10-year longitudinal study, where isolated visual hallucinations predominated early on, with visual plus non-visual hallucinations accounting for progressively higher proportion of experiences as time elapsed [38]. Conversely, some of our studies have demonstrated the presence of non-visual hallucinations in isolation [52–54], whereas multisensory hallucinations without visual involvement seemed rare [54]. Phenomenologically, certain sensory combinations appeared more prevalent, such as comorbidity of visual and tactile components as well as co-occurrence of sensed presence with hallucinations in one or more sensory modalities [55]. Should non-visual or multisensory hallucinations predominate later in PD, we may expect a discrete cognitive profile or more severe cognitive deficits to accompany this. Such a conjuncture was however overturned by Katzen et al. [56], who found no significant cognitive differences between those with visual or multisensory hallucinations, though small subgroup sizes was a major drawback. Yet patterns of clustering of hallucination modalities, along with their timelines in relation to PD illness progression, remain a clinically significant issue to bear in mind.

### Study limitations

The current review was subject to several methodological limitations. Owing to its scope focused on non-visual and multisensory hallucinations, established PD studies centred on visual hallucinations only were excluded (though the number of existing studies likely necessitates a systematic review in its own right). By the same token, research examining hallucinations more broadly without specifying modality were also left out (including those that utilised the UPDRS as their main hallucination assessment tool). Consequently, any findings relating solely to visual hallucinations were incomplete and conclusions drawn from this review must take this into account. The other major limitation relates to lack of consideration of PD medications, especially in terms of its effects and associated timelines. This was a conscious decision, as the range of drugs and varying doses employed to treat a myriad of PD symptoms renders this a complex and somewhat contentious topic. For instance, some PD drugs are known or held to induce psychosis [13, 57, 58], whereas other neuropsychiatric medications are evidently prescribed to treat hallucinations [59, 60]. Until clear effects of these substances on hallucinatory events can be established, current findings will again need to be interpreted within this lens. Moreover, a lack of neuropathological confirmation of clinically suspected PD across the included studies has limited the generalisability of our conclusions, given some cases may be re-classified as dementia with Lewy bodies or some other Parkinsonism syndrome post-mortem. Finally, it is possible that we may have missed papers that presented non-hallucinations data in their full-text, but not the abstract. However, our comprehensive and extensive search strategy, would hopefully have mitigated this. On the other hand, the chief strength of our review is a much needed focus on non-visual and multisensory hallucinations in PD, a hitherto neglected area, despite repeated calls from established PD researchers in the field [38, 61].

### Future research

It is thus clear that future research needs to holistically focus on all hallucination modalities in PD, but especially non-visual and multisensory modes given their relative neglect to date [38, 61], several aspects of which are especially deserving of attention. Evidently, prevalence studies, particularly in auditory, olfactory, somatic-tactile and multisensory (with specified modality combinations) are lacking. Scrutinising descriptive phenomenology of the latter, where thematic and temporal associations are explicitly clarified, will also facilitate a better understanding of what these experiences are like from a patient’s perspective. Given heavy involvement of the visual domain, its intersection with tactile symptoms in so-called ‘string’ hallucinations [62] is of interest, whereas little is known about comorbid olfactory and gustatory hallucinations in PD [34, 46]. Given known olfactory difficulties in the disorder [40] as well as interplay with the gustatory domain [63], this area of enquiry could prove fruitful.

In terms of clinical and cognitive correlates, more targeted studies with regards to non-visual hallucinations are needed, where it is essential to clearly delineate hallucinations by modality. From a practical perspective, achieving ‘pure’ modality subgroups may be impossible, but the next best logical step would be to ensure that these ‘mixed’ modality subgroups are well-documented. Existing studies (even in visual hallucinations) have mostly focused on detecting the presence of hallucinations, but it is equally important to consider what contributes to hallucination severity. Besides the variables considered in this review, there are other clinical correlates for which there is preliminary evidence for further study, including sex, anxiety and even delusions. There is also scope to undertake a more nuanced investigation of intersections between phenomenological facets and cognitive domains. For instance, PD patients with visual hallucinations involving unfamiliar content were found to have more profound deficits in executive inhibition relative to those whose hallucinations comprised recognised content [64]. Such efforts may be replicated considering hallucinations in non-visual modalities. Finally, the few longitudinal studies in the area have been especially enlightening [38, 65]. In this vein, a comprehensive longitudinal examination of hallucination modality in line with PD illness progression will help to address existing gaps in knowledge, and significantly advance the field.

## Conclusions

Even with the limited evidence base analysed in the current review, it is apparent that non-visual and multisensory hallucinations in PD are of clinical significance, and impact a notable proportion of patients. More research attention needs to be devoted to their study, especially in terms of establishing more accurate prevalence rates, as well as elucidating the involvement and clustering of specific non-visual and visual modalities, and their primary clinical-cognitive correlates. Doing so will likely yield prognostic and therapeutic benefits in a bid towards holistic management of the disorder.

## Supplementary Information

Below is the link to the electronic supplementary material.

Supplementary file1 (DOCX 134 KB)

## Data Availability

N/A.
