# Supplementary material for: Non-visual hallucinations in Parkinson’s disease: a systematic review
Source: J Neurol. 2023 Jan 27;270(6):2857–89. doi: 10.1007/s00415-022-11545-6 (PMC10188425; doi:10.1007/s00415-022-11545-6)
Supplement: Supplementary file 1 — Supplementary file1 (DOCX 134 KB) [file 415_2022_11545_MOESM1_ESM.docx]

**Article title:** Non-visual hallucinations in Parkinson’s disease: A systematic review

**Journal name:** Journal of Neurology

**Author names:** Wei Lin Toh, Caitlin Yolland, Caroline Gurvich, James Barnes, & Susan L Rossell

**Corresponding author:** Centre for Mental Health & Brain Sciences, Swinburne University of Technology, [wtoh@swin.edu.au](mailto:wtoh@swin.edu.au)

Table A

*Search syntax employed across PsycINFO APA, PubMed, and Web of Science*

| Database | Syntax |
| --- | --- |
| PsycINFO APA* | (((abstract: (Parkinson*)) AND (abstract: (hallucinat*) OR abstract: (psychosis) OR abstract: (psychotic) OR abstract: (delusion*)) AND (abstract: (Cognit*))) AND ((Any Field: Year: [2020 TO 2022]))) OR (((abstract: (Parkinson*)) AND (abstract: (hallucinat*) OR abstract: (psychosis) OR abstract: (psychotic) OR abstract: (delusion*))) AND ((Any Field: Year: [2020 TO 2022] OR TestYear: [2020 TO 2022])))  (((abstract: (Parkinson*)) AND (abstract: (hallucinat*) OR abstract: (psychosis) OR abstract: (psychotic) OR abstract: (delusion*)) AND (abstract: (Cognit*))) AND ((Any Field: Year: [1970 TO 2020]))) OR (((abstract: (Parkinson*)) AND (abstract: (hallucinat*) OR abstract: (psychosis) OR abstract: (psychotic) OR abstract: (delusion*))) AND ((Any Field: Year: [1970 TO 2020] OR TestYear: [1970 TO 2020]))) |
| PubMed* | ((parkinson*[Title/Abstract]) AND ((((hallucinat*[Title/Abstract]) OR (psychosis[Title/Abstract])) OR (psychotic[Title/Abstract])) OR (delusion*[Title/Abstract]))) OR (((parkinson*[Title/Abstract]) AND ((((hallucinat*[Title/Abstract]) OR (psychosis[Title/Abstract])) OR (psychotic[Title/Abstract])) OR (delusion*[Title/Abstract]))) AND (cogniti*[Title/Abstract])) AND (2020:2022[pdat])  ((parkinson*[Title/Abstract]) AND ((((hallucinat*[Title/Abstract]) OR (psychosis[Title/Abstract])) OR (psychotic[Title/Abstract])) OR (delusion*[Title/Abstract]))) OR (((parkinson*[Title/Abstract]) AND ((((hallucinat*[Title/Abstract]) OR (psychosis[Title/Abstract])) OR (psychotic[Title/Abstract])) OR (delusion*[Title/Abstract]))) AND (cogniti*[Title/Abstract])) AND (1970:2020[pdat]) |
| Web of Science | # 6 – 230 results  #5 OR #4    # 5 – 89 results  #3 AND #2 AND #1    # 4 – 230 results  #2 AND #1    # 3 – 72,845 results  (AB=(cognit*))  AND LANGUAGE: (English) Timespan=1970-2022    # 2 – 5,782 results  (AB=(hallucinat* OR psychosis OR psychotic OR delusion*))  AND LANGUAGE: (English) Timespan=1970-2022    # 1 – 13,782 results  (AB=(Parkinson*))  AND LANGUAGE: (English) Timespan=1970-2022 |

*Note.* *Two-stage search.

Table B

*Prevalence of multisensory hallucinations in Parkinson’s disease (n=17 studies)*

| ***Author, year*** | ***Topic in PD*** | ***Participants; subgroups*** | **Prevalence (%)** | | | | | | | |
| --- | --- | --- | --- | --- | --- | --- | --- | --- | --- | --- |
|  |  |  | ***Any hall*** | ***MSH*** | ***VH*** | ***AH*** | ***OH*** | ***TH*** | ***GH*** | ***SP*** |
| Amar et al., 2014 | Psychosis | 40 | 92.5 | VH+AH+OH+TH =2.5 | 62.5 | 45.0 | 2.5 | 22.5 | - | 27.5 |
| Chacko et al., 1995 | *Clozapine* treatment | 12 | 91.7 | - | 83.3 | 66.7 | 8.3 | 16.7 | - | - |
| Dudley et al., 2019 | MSH in context of VH | 41 | - | MSH=10.3; VH+AH=2.6, VH+TH=7.7, VH+OH/GH =2.6 | 89.7 | - | - | - | - | - |
| Fernandez et al., 1992 | Hall and motor symptoms | 50 | 60.0 | VH+AH=18.0 | 58.0 | - | - | - | - | - |
| Grossi et al., 2005 | Hall (without dementia) | 48 | 29.2 | VH+AH=6.3 | VH_only_=18.8 | AH_only_=4.2 | - | - | - | - |
| Gupta et al., 2004 | Hall | 43 | 34.9 | - | 25.6 | 14.0 | - | - | - | 7.0 |
| Haeskedewick, 1995 | Hall | 36 | 44.4 | VH+AH=8.3 | 25.0 | 5.6 | 5.6 | 16.7 | 0 | - |
| Marques et al., 2021 | MSH in context of VH | 28 | - | VH+AH=21.4, VH+OH=17.9, VH+SP=29.4 | - | - | - | - | - | - |
| Mellers et al., 1995 | *Apomorphine* treatment | 24; PD_psychosis_ =16 | 58.3; 87.5 | - | 45.8; 68.8 | 16.7; 25.0 | - | 12.5; 18.8 | - | - |
| Moskovitz et al., 1978 | Levodopa-induced psychosis | 31 | 29.5 | VH+AH=25.8 | VH_only_=61.3 | AH_only_ =9.7 | - | TH_only_=4.8 | - | - |
| Pagonabarraga et al., 2016 | SP in drug naïve PD | 50 | - | - | 4.0 | 2.0 | 4.0 | - | - | 14.3 |
| Pagonabarraga et al., 2014 | Neural correlates of SP | 46 | - | - | - | - | - | - | - | 17.4 |
| Rabey et al., 1995 | *Clozapine* treatment | 27 | - | - | 63.0 | 11.8 | - | - | - | - |
| Reckner et al., 2020 | SP | 18 | - | VH+SP=33.3 | 33.3 | - | - | - | - | 100.0 |
| Wada-Isoe et al., 2008 | Hall scale validation | 41 | 34.1 | VH+AH=9.8, VH+cenesthetic= 4.9, VH+AH+TH=2.4 | 31.7; VH_only_=14.6 | AH_only_ =2.4 | - | - | - | - |
| Whitehead et al., 2008 | Halls and circadian rhythm | 50 | 54.0 | VH+AH=2.0, VH+SP=2.0, TH+SP=2.0 | 30.0 | 8.0 | 6.0 | 8.0 |  | 6.0 |
| Yoshida et al., 2009 | Brain stimulation in hall | 18 | 72.2 | - | 44.4 | 22.2 | - | - | - | 16.7 |

*Note.* PD=Parkinson’s disease; Hall=hallucinations; MSH=multisensory hallucinations, broken down into modalities where possible; VH=visual hallucinations; AH=auditory hallucinations; OH=olfactory hallucinations; TH=tactile hallucinations; GH=gustatory hallucinations; SP=sensed presence, also referred to as “Guardian angel”, “feeling of presence” or “extracampine hallucinations”.

Table C

*Select clinical and cognitive correlates of hallucinations in Parkinson’s disease (n=13 studies)*

| **Author, year** | **Participant subgroup/ numbers** | **Clinical correlates** | | | | |  | **Cognitive correlates** |
| --- | --- | --- | --- | --- | --- | --- | --- | --- |
|  |  | ***Age (years)*** | ***Age at PD onset*** | ***Length of illness (years)*** | ***PD severity***  ***(******HY or MDS-UPDRS)*** | ***Major depressive disorder*** | ***Dementia*** |  |
| Aarsland et al., 2001 | PD_hall+_=38 vs PD_hall-_=93 | ns | ns | - | HY: PD_hall+_>PD_hall-_, *p*<.001 | SBMD: ns | PD_hall+_>PD_hall-_, *p*<.001 | MMSE: PD_hall+_<PD_hall-_, *p*<.001 |
| Benbir et al., 2006 | PD_hall+_=70 vs PD_hall-_=60 | ns | - | ns | UPDRSI: PD_hall+_>PD_hall_, *p*<.001  HY, UPDRSII, UPDRSIII: ns | - | - | - |
| de Maindreville et al., 2005 | PD_hall+_=18 vs PD_hall-_=55 (longitudinal) | ns | ns | - | HY: PD_hall+_>PD_hall_, *p*=.019  UPDRSI: PD_hall+_>PD_hall_, *p*=.049  UPDRSII: PD_hall+_>PD_hall_, *p*=.040 | CES-D: PD_hall+_>PD_hall_, *p*=.003 | ns | - |
| Factor et al., 2014 | PD_hall+_=25 vs PD_hall-_=96 (also ^ꝉ^PD_del_ not included) | ns | ns | ns | UPDRSIII: ns | SCID: ns | - | ns for MMSE, attention, language, executive function, memory, and visuospatial abilities |
| Fernandez et al., 1992 | PD_hall+_=30 vs PD_hall-_=20 | PD_hall+_>PD_hall_, *p*<.001 | - | ns | HY: ns | - | - | MMP: PD_hall+_>PD_hall_, *p*<.001 |
| Grossi et al., 2005 | PD_hall+_=14 vs PD_hall-_=34 | ns | - | PD_hall+_>PD_hall_, *p*=.02 | HY: ns | - | - | Phonological fluency: PD_hall+_<PD_hall_, *p*=.004;  semantic fluency: PD_hall+_<PD_hall_, *p*=.001;  immediate free recall: PD_hall+_<PD_hall_, *p*=.032; ns for MMSE, RCPM and delayed free recall |
| Gupta et al., 2004 | PD_hall+_=15 vs PD_hall-_=28 | ns | - | PD_hall+_>PD_hall_, *p*<.05 | HY: PD_hall+_>PD_hall_, *p*<.05  UPDRSII, UPDRSIII: ns | BDI-II: ns | - | - |
| Haeskedewick, 1995 | PD_hall+_=16 vs PD_hall-_=20 | PD_hall+_>PD_hall_, *p*<.05 | - | PD_hall+_>PD_hall_, *p*<.01 (when controlled for age) | HY: PD_hall+_>PD_hall_, *p*<.02 | GDS: PD_hall+_>PD_hall_, *p*<.05 (ns when controlled for age) | - | MMSE: PD_hall+_<PD_hall_, *p*<.02; verbal fluency: PD_hall+_<PD_hall_, *p*<.02 |
| Ikeda et al., 2016 | PD_hall+_=31 vs PD_hall-_=32 | ns | - | ns | HY: ns  UPDRS: PD_hall+_>PD_hall-_, *p*=.034 | SDS: ns | - | MMSE: ns |
| Leu-Semenescu et al., 2011 | PD_hall+_=26 vs PD_hall-_=74 | ns | ns | ns | UPDRSIII:  PD_hall+_>PD_hall-_, *p*=.003 | ns | - | ns |
| Paleacu et al., 2005 | PD_hall+_=76 vs PD_hall-_=158 | PD_hall+_>PD_hall-_, *p*<.001 | - | ns | HY: PD_hall+_>PD_hall-_, *p*<.001 | - | PD_hall+_>PD_hall-_, *p*<.001 | MMSE: PD_hall+_<PD_hall-_, *p*<.001 |
| Papapetropoulos et al., 2008 | PD_hall+_=31 vs PD_hall-_=39 | ns | ns | ns | HY, UPDRS: ns | ns | - | MMSE: ns |
| Whitehead et al., 2008 | PD_hall+_=27 vs PD_hall-_=23 | ns | ns | ns | UPDRSIII: PD_hall+_>PD_hall-_ *p*<.01; UPDRSIV: ns | - | - | MMSE: PD_hall+_<PD_hall-_, *p*<.01 |

*Note.* PD=Parkinson’s disease; PD_hall+_=PD with hallucinations; PD_hall-_=PD without hallucinations; PD_del_=PD with delusions; HY=Hoehn-Yahr scale; UPRDRS=Movement Disorders Society Unified Parkinson Disease Rating Scale; ns=not significant; SBMD: Scale for Brief and Major Depression; MMSE=Mini Mental State Examination; CES-D=Centre for Epidemiological Studies-Depression Scale; SCID=Structured Clinical Interview for DSM; RCPM=Raven’s Coloured Progressive Matrices; GDS=Geriatric Depression Scale; TICS=Telephone Interview for Cognitive Status.

^ꝉ^ This group comprised individuals with delusions, but with or without hallucinations, the former of whom were not included in the hallucinations subgroup.

Table D

*Significant associations or predictors for hallucinations in Parkinson’s disease (n=8 studies)*

| **Author, year** | **Participant age (years), sex (% male), numbers** | **Significant associations or predictors** | **Covariates (if any)** | **Statistics (*r* or OR, CI, *p*)** | **Variable of interest** |
| --- | --- | --- | --- | --- | --- |
| Aarsland et al., 2001 | 74.3±7.3, 43, 131 | Dementia  PD severity (HY≥3) | - | OR=3.9, CI=1.5-10.4^#^  OR=3.4, CI=1.0-12.0^#^ | Presence of hallucinations |
| Chou et al., 2005 | 71.7±7.4, 68, 160 | Clinical Global Impression | Age, HY, MMSE | *r*=.45-53*** | Hallucination subscale/item (BPRS/NPI) |
| de Maindreville et al., 2005 | 68.4±9.8, na, 127 | Ocular disorders  Motor impairment (axial)  Severe sleep disturbance | - | OR=14.3, CI=2.5-80.9, *p*=.009  OR=9.1, CI=1.6-52.0, *p*=.019  OR=5.7, CI=1.2-27.4, *p*=.001 | Presence of hallucinations |
| Ikeda et al., 2016 | 69.8±7.8, 41, 63 | Fatigue (BFI) | MDS-UPDRS | OR=1.0, CI=1.0-1.1, *p*=.045 | First onset hallucinations |
| Leu-Semenescu et al., 2011 | 63.5±10.9, 66, 100 | PD severity (UPDRSIII)  Sleepiness (ESS) | - | OR=1.1, CI=1.0-1.1, *p*=.002  OR=1.1, CI=1.0-1.3, *p*=.004 | Presence of hallucinations |
| Paleacu et al., 2005 | 76.0±11.0, 29, 276 | Cognition (MMSE)  Familial dementia history | - | na, na, *p*<.001  na, na, *p*=.001 | Presence of hallucinations |
| Svetel et al., 2012 | 62.3±9.3, 53, 95 | Length of illness  Neuropsychiatric symptoms (NPI) | - | OR=8.4, 2.4-19.4, *p*=.024  OR=11.3, 3.6-34.7, *p*=.002 | Presence of hallucinations |
| Wada-Isoe et al., 2008 | 69.4±9.5, 24, 41 | Cognition (MMSE)  PD severity (HY) | - | β=-0.4, na, *p*=.016  β=0.3, na, *p*=.030 | Severity of hallucinations |

*Note.* OR=odds ratio; CI=95% confidence interval; na=not available; PD=Parkinson’s disease, HY=Hoehn-Yahr scale; UPRDRS=Movement Disorders Society Unified Parkinson Disease Rating Scale; MMSE=Mini Mental State Examination; BPRS=Brief Psychiatric Rating Scale; NPI=Neuropsychiatric Inventory; BFI=Brief Fatigue Inventory; ESS=Epworth Sleepiness Scale.

^#^Significant, but no *p* value provided, ****p*<.001.

Table E

*Risk of bias assessment for case reports/series using JBI-CAC (n=30 studies)*

| **Author, year** | ***Case study or series*** | ***1. Was patient demographic characteristics clearly described?*** | ***2. Was patient history clearly described, with timeline?*** | ***3. Was patient PD presentation clearly described?*** | ***4. Were diagnostic tests for PD clearly described?*** | ***5. Were assessment methods for hallucinations adequate?*** | ***6. Was information descriptive enough for phenomenology?*** | ***Total scoring (out of a maximum of 12)*** |
| --- | --- | --- | --- | --- | --- | --- | --- | --- |
| Abe et al., 2016  Arnulf et al., 2000  Clark, 1998  Dashtipour et al., 2021  Ergun et al., 2009  Factor & Molho, 2004  Fenelon et al., 2000^ꝉ^  Fenelon et al., 2002  Gondim Fde et al., 2010  Jimenez-Jimenez et al., 1997  Kataoka et al., 2014  Kataoka & Ueno, 2014  Kesserwani, 2021  Landis & Burkhard, 2008  Lenka et al., 2018  McAuley & Gregory, 2012  Meco & Bernardi, 2007  Mittal & Giron, 2010  Muralidharan et al., 2011  Nagata et al., 2013  Nishioka et al., 2014  Onofrj et al., 2000  Parsa & Bastani, 1998  Perez-Perez et al., 2016  Potheegadoo et al., 2022  Roberts et al., 1989  Ting et al., 2019  Tousi & Frankel, 2004  Voon & Lang, 2004  Wand, 2012 | Study  Study  Study  Study  Study  Series  Series  Series  Series  Study  Study  Study  Study  Series  Study  Series  Study  Study  Study  Study  Study  Study  Series  Series  Series  Study  Study  Study  Study  Study | 2  2  2  2  2  2  2  2  2  2  2  2  2  2  2  2  2  2  2  2  2  2  2  2  2  2  1  2  2  2 | 2  2  2  2  2  2  2  0  2  2  2  2  2  2  2  2  2  2  2  2  2  2  2  0  1  2  2  2  2  2 | 2  2  2  2  2  2  2  2  2  2  2  2  2  2  2  2  2  2  2  2  2  2  2  2  2  2  2  2  2  2 | 2  2  1  2  2  2  2  2  2  2  2  2  2  0  2  2  2  2  2  2  2  2  2  2  2  2  2  2  2  2 | 1  1  1  1  1  1  1  1  1  1  1  1  1  1  1  2  2  1  2  1  1  1  2  1  2  1  1  1  2  1 | 2  2  2  2  2  2  2  2  2  2  2  2  2  1  2  2  2  2  0  2  1  2  1  2  2  1  2  2  2  1 | 11  11  10  11  11  11  11  9  11  11  11  11  11  8  11  12  12  11  10  11  10  11  11  9  11  10  10  11  12  10 |

*Note.* JBI-CAC=JBI Critical Appraisals Checklist for Case Studies; PD=Parkinson’s disease. For all six items, 2=Yes, 1=Maybe or unclear, 0=No.

^ꝉ^Also a group design study.

Table F

*Risk of bias assessment for group design studies using NOQAS (n=62 studies)*

| **Author, year** | **Is case definition (i.e. diagnostic criteria) adequate? *(2=Yes, PD diagnosis with independent validation; 1=Yes, PD diagnosis based on record linkage or self-report; 0=No description of PD diagnosis)*** | **Representative-ness of cases *(1=Truly or somewhat representative of average in target PD population; 0=Potential for selection bias or not stated)*** | **Selection of controls *(2=Yes, control group selected; 1=not applicable, or control group not relevant to research questions; 0=No control group or no description)*** | **Definition of controls *(2=Both clinical and non-clinical controls; 1=Either clinical or non-clinical controls; 0=No control group, or no description)*** | **Comparability of cases and controls on basis of design or analysis *(1=Matches cases and controls in design or analysis; 0=Does not match groups or no description)*** | **Validity of hallucinations assessment *(2=Gold standard or validated measure; 1=Clinician rating or unvalidated measure; 0=Other non-standardised measure or no description)*** | **Total scoring (out of a maximum of 10)** |
| --- | --- | --- | --- | --- | --- | --- | --- |
| Aarsland et al., 2001  Amar et al., 2014  Bannier et al., 2012  Barrett et al., 2017  Benbir et al., 2006  Chacko et al., 1995  Chendo et al., 2021  Chou et al., 2005  de Chazeron et al., 2015  de Maindreville et al., 2005  Dudley et al., 2019  Factor et al., 2014  Fenelon et al., 2000^ꝉ^  Fenelon et al., 2011  Fenelon et al., 2010  Fernandez et al., 1992  Goetz et al., 2011  Goetz et al., 1982  Goetz et al., 1998  Grossi et al., 2005  Gupta et al., 2004; Haeskedewick, 1995  Holroyd et al., 2001  Ikeda et al., 2016  Inzelberg et al., 1998; Kataoka & Ueno, 2015  Katzen et al., 2010  Kulick et al., 2018  Lee & Weintraub, 2012  Lenka et al., 2017  Leu-Semenescu et al., 2011  Llorca et al., 2016  Mack et al., 2012  Marques et al., 2021  Marsh et al., 2004  Matsui et al., 2007  Mellers et al., 1995  Moskovitz et al., 1978  Muller et al., 2018  Nishio et al., 2017  Omoto et al., 2021  Pacchetti et al., 2005  Pagonabarraga et al., 2016  Pagonabarraga et al., 2014  Paleacu et al., 2005  Papapetropoulos et al., 2008  Rabey et al., 1995  Rai et al., 2015  Rana et al., 2013  Reckner et al., 2020  Sawada et al., 2013  Shine et al., 2015  Solla et al., 2021  Svetel et al., 2012  Trosch et al., 1998  Wada-Isoe et al., 2008  Whitehead et al., 2008  Wood et al., 2015  Yoshida et al., 2009  Zhang et al., 2021  Zhong et al., 2021  Zhu et al., 2017 | 2  2  2  2  2  1  2  2  2  2  2  2  2  1  2  2  2  1  1  2  2  2  2  2  1  2  2  2  2  2  2  2  2  2  2  2  2  1  2  2  2  2  2  2  2  2  2  2  1  1  1  2  2  2  1  2  2  1  1  2  2  2 | 1  1  1  1  1  1  1  1  0  1  1  1  1  1  1  1  1  1  1  1  1  1  1  1  1  1  1  1  1  0  1  1  0  0  1  0  0  1  0  0  1  1  0  0  0  0  0  1  1  1  1  1  1  1  1  0  0  1  0  1  1  1 | 2  1  2  2  2  1  2  1  1  2  1  2  2  2  1  2  1  1  2  2  2  1  1  2  2  1  2  2  1  1  2  1  1  1  2  2  2  1  1  1  1  1  2  2  2  2  1  2  2  2  1  2  2  2  0  2  2  2  1  2  2  2 | 1  0  2  1  1  0  1  0  0  1  0  1  1  1  0  1  0  0  1  1  1  0  0  1  1  0  2  1  0  0  1  0  0  0  2  1  2  0  0  0  0  0  1  1  1  1  0  1  1  1  0  1  2  1  0  1  2  1  0  2  1  1 | 1  0  1  1  1  0  1  0  0  1  0  1  1  1  0  1  0  0  1  1  1  0  0  1  1  0  1  1  0  0  1  0  0  0  1  1  1  0  0  0  0  0  1  0  1  1  0  1  1  1  0  1  1  1  0  1  1  1  0  1  1  1 | 2  2  1  2  1  2  2  2  2  1  2  2  1  0  1  1  2  1  1  1  1  1  1  1  1  1  2  2  2  1  1  2  1  2  2  0  1  1  2  2  1  2  2  2  1  1  0  2  2  1  1  1  2  1  1  2  1  1  2  2  1  2 | 9  6  9  9  8  5  9  6  5  8  6  9  8  7  5  7  5  4  7  8  8  5  5  8  7  5  10  9  6  4  8  6  4  5  10  6  8  4  5  5  5  6  8  7  7  7  3  9  8  7  4  8  10  8  3  8  8  7  4  10  8  9 |

*Note.* NOQAS=Newcastle-Ottawa Quality Assessment Scale for Case Control Studies; PD=Parkinson’s disease.

^ꝉ^Also a case series.
